# Integrated 3D Printing of Liquid Metal and Elastomer for Soft Robots and Electronics

**DOI:** 10.34133/research.1174

**Published:** 2026-03-06

**Authors:** Xiaoyu Song, Mengfan Zhang, Xiaoyong Zhang, Zhihao Lv, Shaoxing Qu, Guoyong Mao

**Affiliations:** State Key Laboratory of Fluid Power and Mechatronic Systems, Key Laboratory of Soft Machines and Smart Devices of Zhejiang Province, Center for X-mechanics, Department of Engineering Mechanics, Zhejiang University, Hangzhou 310027, People’s Republic of China.

## Abstract

Soft robots and stretchable electronics, typically composed of stretchable elastomers and embedded conductive coils, have been widely investigated for applications in actuation, sensing, and communication. However, their fabrication still relies heavily on multistep and labor-intensive conventional methods. Here, we present a multimaterial 3-dimensional (3D) printing strategy based on direct ink writing technology, which enables the one-step fabrication of stretchable elastomers embedded with high-conductivity multilayer coils. This is achieved by alternately printing elastomer and nickel-particle-modified liquid metal (NLM) coil layers in a program-controlled sequence, with vertically printed NLM cones connecting adjacent NLM layers. With this strategy, we achieved one-step fabrication of a 4-layer-coil soft electromagnetic actuator (SEMA) and a self-sensing SEMA integrating sensing and driving modules, without the need for manual bonding or post-processing. We further built 3 functional devices to show the potential applications of this integrated 3D printing strategy: a sensor-integrated soft gripper capable of perceiving its own grasping state, a bio-inspired manta-like soft electromagnetic robot that achieves a swimming speed of 29 mm/s, and a SEMA integrated with a Hall sensor and a red light-emitting diode, which exhibits strong mechanical robustness. Overall, the integrated 3D printing strategy not only simplifies the fabrication but also enables the multifunctional and miniaturized design of soft robots and electronics.

## Introduction

Soft robots and stretchable electronics have undergone rapid development in recent years, owing to their adaptability to diverse environments and safe interaction with humans [1–12]. These characteristics have enabled their widespread applications, ranging from minimally invasive medical devices and wearable electronics to exploration robots. Among various types of soft robots, electromagnetic soft robots stand out due to their unique advantages, such as remote actuation, rapid responsiveness, and versatile motion under external magnetic fields [13–19]. A typical structure of soft electromagnetic robots (SEMRs) consists of stretchable bodies embedded with liquid metal (LM) coils, featuring high electrical conductivity and excellent elasticity [20]. Leveraging LM coils and Laplace-force actuation, this structural design enables programmable force distribution, dynamic responses, and reconfigurable functionalities, thereby offering a promising pathway for next-generation soft electromechanical systems.

However, conventional fabrication methods for SEMRs and stretchable electronics are still limited by process complexity and labor intensity. For example, the molding method, which involves casting elastomers in prefabricated molds, assembling elastomer components to form sealed channels, and finally injecting LM with a syringe, has been widely used for fabricating LM-elastomer-based devices [20–23]. Although mature and cost-effective, this process involves multiple steps and often results in defects such as bubbles or cracks within the elastomer matrix. Another commonly used method is the sacrificial template method, where a solid template of the desired coil geometry is embedded in the uncured elastomer and subsequently removed after curing to form hollow channels for LM infusion [24,25]. While this method allows the fabrication of multilayer structures, it is limited by process complexity, the high cost of sacrificial materials, and the risk of incomplete template removal.

Unlike the above-mentioned conventional fabrication methods, various 3-dimensional (3D) printing technologies offer a streamlined, mold-free process that enables direct construction of programmable coils and complex soft structures (Table S1). Many researchers have achieved impressive advances in 3D printing soft electronics using other conductive materials (e.g., silver paste, conducting polymers, and carbon-based conductors) [26–29]. These materials, however, exhibit relatively low electrical conductivity or limited stretchability compared to that of LM, rendering them unsuitable for high-power operational scenarios of soft robots like SEMRs. Furthermore, most investigations only focus on the printing of conductors or the printing of elastomeric structures rather than a full monolithic fabrication [30–37]. These studies have made meaningful progress, laying the foundation for fully integrated fabrication of LM-elastomer soft electromagnetic actuators (SEMAs) with multilayer coil architectures. Recently, some researchers have attempted fully 3D printing approaches. Lin et al. [38] developed an LM-Carbopol hydrogel emulsion ink and demonstrated multimaterial printing by alternately depositing this ink and a polydimethylsiloxane (PDMS)–polytetrafluoroethylene (PTFE) insulating matrix, resulting in composite structures. However, owing to the incorporation of hydrogel, the printed coils exhibited relatively low conductivity and required external activation to achieve electrical conduction. Zhang et al. [39] proposed a coaxial-nozzle-based printing strategy that enables one-step fabrication of LM cores encapsulated in elastomeric sheaths. Although this approach enables the construction of 3D geometries, the volume density of LM coils is constrained, as the overall architecture relies on the linear stacking of elastomer-sheathed LM wires. This reliance limits the design flexibility and integration needed for advanced SEMRs.

Until now, owing to the high surface tension and liquid-like fluidity of LM, the monolithic printing of soft elastomers embedded with multilayer LM coils remains a key challenge. Here, we present a multimaterial 3D printing strategy based on a direct ink writing (DIW) printer that enables integrated fabrication of stretchable elastomers embedded with multilayer NLM coils. In the printing, 3 different inks are employed: NLM, Ecoflex (ECO) (a platinum-catalyzed addition-cure silicone elastomer), and a custom-formulated elastomer composite, named ECO-PDMS-PTFE composite (EPPC), which is formulated by mixing ECO, PDMS solution, and PTFE powders. EPPC ink is first printed to form the substrate and peripheral structures, followed by the deposition of NLM ink to form the first conductive coil layer. Successive ECO and NLM layers are then alternately printed, thereby constructing the multilayer architecture. At the end of each coil layer, an NLM cone is printed vertically to establish interconnections between adjacent coil layers, forming continuous current pathways across multiple layers. This integrated printing process eliminates the need for prefabricated molds, template removal, or post-infusion, thereby enabling DIW of stretchable elastomer structures embedded with multilayer conductive coils. The successful realization of this process depends on 5 key factors, summarized as follows:

1. The NLM must exhibit suitable yield stress and viscoelasticity, allowing it to achieve vertical printing and thus form interlayer connections.
2. The printed NLM wires could maintain their filamentary shape without collapsing or retracting under surface tension, to facilitate stable and continuous coils.
3. Despite incorporating additional particles, the NLM retains high electrical conductivity, which is essential for minimizing resistive losses during operation.
4. The EPPC ink, which forms the substrate and peripheral structures, is expected to possess adequate self-supporting capability, thereby preserving structural fidelity.
5. Each printed elastomer layer must cure with high surface uniformity, thus enabling the consistency of NLM deposited on subsequent layers.

Fulfilling these requirements, our approach achieves one-step fabrication of multilayer SEMRs and soft electronics, opening opportunities for advanced soft robotic systems with complex architectures.

## Results

### Design principle of SEMRs

**Fig. 1.**
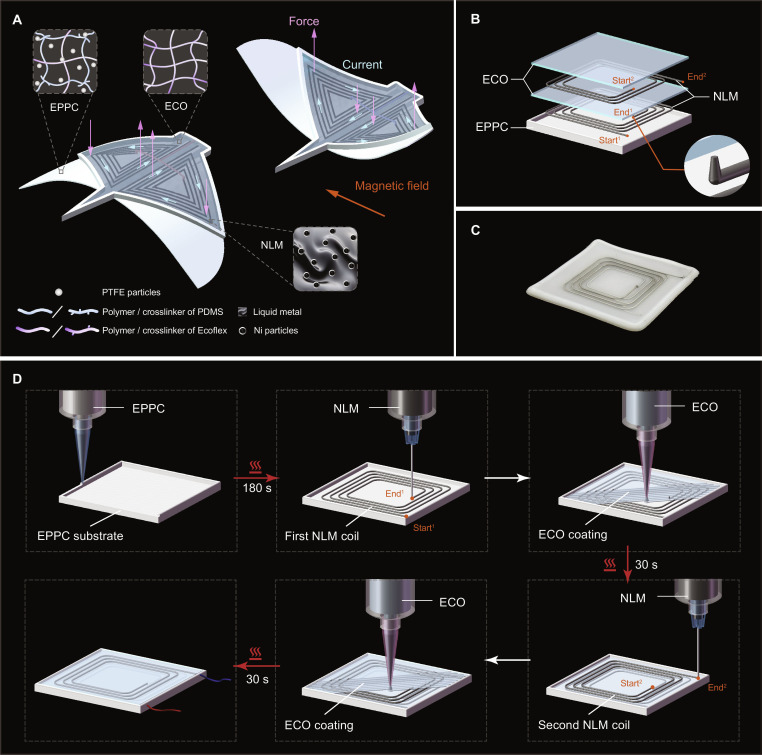
Design principle of SEMRs and schematics of the multimaterial 3D printing process. (A) Schematic illustration of the materials and actuation principle of SEMRs using the Manta-like SEMR as an example. (B) Exploded view of the square-shaped SEMA with a double-layer coil, comprising 1 EPPC substrate, 2 NLM layers, and 2 ECO layers. (C) Photograph of the printed double-layer SEMA. (D) Step-by-step illustration of the integrated multimaterial 3D printing process for constructing multilayer SEMAs. The red arrows denote the curing process of the printed layer.

Figure 1A illustrates the design principle of SEMRs, including both their driving strategy and manufacturing materials. A bio-inspired manta-like SEMR consists of multiple elastomer layers and embedded multilayer coils. Its actuation relies on the Laplace force, as shown in Fig. S1, a mechanical force when a current-carrying conductor is placed within an external magnetic field. The direction and magnitude of the Laplace force are governed by F=I∫dl×Β, where **I** is the current vector passing through the wire, dl is an infinitesimal segment of the wire, and **B** is the magnetic flux density vector [20]. Based on this principle, we designed the LM coils in a manta-like SEMR by leveraging the multimaterial 3D printing strategy to replicate both the morphological and kinematic features of batoid fishes. Mantas are renowned for their stable swimming and periodic flapping-gliding patterns, which are achieved through the complex deformation of their broad pectoral fins [40]. By programmatically depositing elastomers and conductive NLM inks, we constructed a monolithic structure that mimics the profile of a manta while embedding multilayer NLM coils within the fins to serve as artificial muscles. When placed in a uniform magnetic field, the strategic arrangement of these embedded coils allows the SEMR to experience an instantaneous reversal of the Laplace force upon switching the current direction. This force drives the fins of manta-like SEMR up and down, generating the rhythmic kinematics required for stable underwater propulsion.

This manta-like SEMR is fabricated using 3 different printing inks (Fig. 1A). The first ink is the custom-formulated elastomer EPPC, which is used to construct the substrate and peripheral structures. Benefiting from its optimized rheological properties, EPPC exhibits excellent self-supporting capability, ensuring structural integrity throughout the printing process. The second ink is ECO, which is employed for the upper layers. It is deposited within the surrounding EPPC borders, where its high fluidity facilitates self-leveling and leads to the formation of smoother and more uniform elastomer layers. After being deposited onto a heating platform, both EPPC and ECO undergo crosslinking and curing, transforming from printable solutions into solid and elastic elastomers. The third is NLM, a modified LM ink prepared by dispersing nickel particles into LM. Owing to its high conductivity and printability, it can be directly printed onto cured elastomers, thereby forming embedded conductive coils in SEMAs. The NLM ink remains liquid at room temperature, providing stretchable conductivity that accommodates structural deformation during operation.

### Schematics of the multimaterial 3D printing process

To illustrate the integrated 3D printing process of SEMAs, a square-shaped actuator with a double-layer coil is selected as a representative model (structural parameters are in Fig. S2). As shown in the exploded view (Fig. 1B), this SEMA consists of 1 EPPC substrate, 2 NLM layers, and 2 ECO layers. A photograph of a printed sample is shown in Fig. 1C, while its printing process is illustrated in Fig. 1D. First, the EPPC is printed onto the heating platform to form the substrate and the peripheral of the SEMA. The printed EPPC is cured at 60°C on a heating platform and is simultaneously irradiated by an infrared lamp at a distance of 1 cm for 180 s. Following this, the first NLM coil layer is printed onto the cured elastomer substrate. At the end of the first coil layer, a vertical NLM cone is printed at a speed of 5 mm/s to establish electrical connectivity with the subsequent coil layer. Then, the ECO is printed to cover the first coil, excluding the apex of the cone. The underlying coil and the connecting cone maintain their geometric integrity without collapse or deformation, owing to the improved self-supporting properties of the NLM. The ECO layer is cured via the same procedure as the EPPC layer, but with a marked shorter duration of 30 s irradiated by the infrared lamp. Subsequently, the second NLM layer is printed, starting from the exposed connecting cone of the previous layer. Finally, a top ECO layer is printed to encapsulate the NLM coils. Ultimately, a SEMA with a double-layer coil structure is obtained. The in situ 3D printing video is provided in Movie S1. Detailed printing parameters, including nozzle types, nozzle diameters, printing pressures, and printing speeds, are listed in Table S2. Printing and curing times are summarized in Table S3.

For multilayer structures, their fabrication principle is consistent with that of the SEMA with a double-layer coil. Additional layers are formed by alternately depositing NLM and ECO covers, following the same procedure as described above. Since the printing and curing times of ECO are substantially shorter than those of EPPC (Table S3), the fabrication of additional layers consumes considerably less time than that of the substrate layer. Notably, adjacent layers are interconnected through vertically printed NLM. Consequently, except for the last coil layer, NLM cones are printed at the coil ends of all other layers. Besides, the surface roughness characterization of the cured elastomers (Fig. S3) confirms refined surface morphologies, allowing successful deposition of subsequent NLM. Overall, this approach enables 3D printing of SEMAs with multilayer coil architectures.

### Mechanical properties of elastomers

**Fig. 2.**
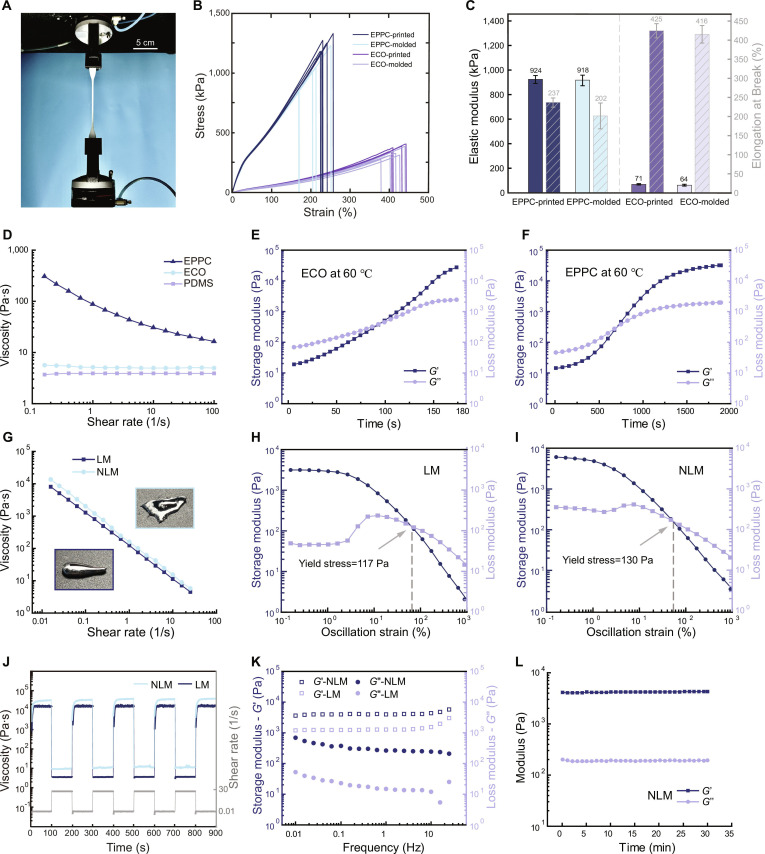
Mechanical and rheological characterization of elastomers and LM inks. (A) Tensile test setup for cured elastomer samples. (B) Stress–strain curves of printed and molded EPPC and ECO. (C) The elastic modulus and the elongation at break of the printed and molded EPPC and ECO. (D) Viscosity–shear rate curves of elastomer inks. (E and F) Time-dependent evolution of storage modulus (*G*′) and loss modulus (*G*″) for ECO and EPPC inks during curing at 60 °C. (G) Viscosity comparison and photographs of LM and NLM inks. (H and I) Oscillatory amplitude sweep of LM and NLM inks. (J) Three-interval thixotropy tests for characterizing viscosity recovery behaviors of LM and NLM. (K) Frequency sweep results of LM and NLM showing *G*′ and *G*″. (L) Time-dependent stability of NLM ink, represented by *G*′ and *G*″.

To characterize the mechanical performance of ECO and EPPC elastomers, tensile tests were performed (Fig. 2A). Figure 2B and C present the stress–strain curves, elastic moduli, and elongations at break of these materials, with each elastomer fabricated via both molding and 3D printing to facilitate comparative analysis. The printed EPPC exhibits an average elastic modulus of 924 kPa and an elongation at break of 237%, which are nearly comparable to the mold-cast version (918 kPa and 202%, respectively). For ECO, the printed samples display an average elastic modulus of 71 kPa and an elongation at break of 425%, demonstrating high consistency with mold-cast samples (64 kPa and 416%, respectively). These results demonstrate that the mechanical properties of 3D-printed specimens are highly consistent with molded specimens, with statistically insignificant differences (*P* > 0.05). Minor performance advantages in printed specimens are attributed to the more uniform distribution of liquid-state elastomer inks during the DIW process compared to that of manual casting for mold-based specimens, which may occasionally entrap microbubbles or induce subtle material inhomogeneities.

Furthermore, to evaluate the adhesion stability of the multilayer structure, T-peel tests were conducted (Fig. S4). Three groups of specimens were prepared, namely, EPPC–EPPC, EPPC–ECO, and ECO–ECO. The adhesion energies of all interfaces are in the range of 350 to 850 J/m^2^, which demonstrates satisfactory adhesion performance sufficient for practical applications in SEMRs and soft electronics (details in the Supplementary Text).

### Rheological properties of printing inks

Only printing inks with suitable thixotropic properties are compatible with DIW 3D printing. Specifically, such inks should exhibit shear thinning during extrusion to ensure printability and rapidly recover their high viscosity after deposition to maintain shape fidelity [41]. To evaluate the shear-thinning behavior of the prepared inks, steady-state flow measurements were performed using a rotational rheometer. As shown in Fig. 2D, pure PDMS and ECO inks exhibit minimal variation across the tested shear rates. In contrast, the EPPC ink displays an initial viscosity of 306 Pa·s (0.16 s^−1^), 2 orders of magnitude higher than that of the other 2 inks, and demonstrates clear shear-thinning characteristics. This indicates that the addition of PTFE renders the silicone-based ink highly compatible with DIW printing and simultaneously enhances its viscosity under quasi-static conditions, which confirms its superior shape retention capability.

Figure 2E and F illustrate the time-dependent modulus variations of ECO and EPPC inks at 60 °C. After deposition on the heated printing platform, the inks undergo gradual solidification with similar trends. Initially, the storage modulus (*G*′) is lower than the loss modulus (*G*″). Over time, both moduli increase substantially, with *G*′ eventually surpassing *G*″, indicating the progressive curing of the inks. The gelation times (defined as the time when *G*′ = *G*″) for ECO and EPPC inks are 93 and 707 s, respectively. In fact, during standard printing, in addition to the heated platform, infrared lamp irradiation is additionally applied, which yields a shorter solidification time than that observed in rheological tests.

As for printing conductive coils, the pristine LM ink is ill-suited due to its inherent high surface tension and liquid-like fluidity [42]. Therefore, tailoring the rheological properties of LM is indispensable to achieve high-precision extrusion and excellent shape retention during printing. Building on the mechanism reported in the literature [32], we incorporated nickel particles into the LM matrix via mechanical stirring and ultrasonication, after which the nickel particles achieved a highly dispersed state within the LM matrix [43]. The addition of nickel particles increases the solid content, producing a thickening effect. These in situ formed oxides act as a “scaffold” that stabilizes the suspension of nickel particles and inhibits their sedimentation. Furthermore, as reported in the literature [43], interfacial chemical reactions occur among Ni, Ga, and In, yielding intermetallic compound solid phases (Ga_4_Ni_3_ and InNi_3_) during the mixing process. Consequently, the NLM ink undergoes a distinct transformation from a glossy, highly fluid state to a denser, more viscous paste (insets in Fig. 2G). The initial viscosity of NLM reaches 1.4 × 10^4^ Pa·s (0.016 s^−1^), which is substantially higher than that of LM (8.2 × 10^3^ Pa·s, 0.016 s^−1^). This distinct shear-thinning behavior is critical for printing: at low shear rate, the ink exhibits high viscosity, which is essential for maintaining shape fidelity. At high shear rate (25.1 s^−1^), the viscosity drops to 5.8 Pa·s, facilitating smooth extrusion through nozzles.

Figure 2H and I characterize the linear viscoelastic region (LVR) and yield stress of the LM and NLM inks via amplitude sweep experiments. Within the LVR (low strain region), both inks exhibit a solid-like behavior where *G*′ exceeds *G*″, maintaining a stable plateau. Notably, the NLM ink exhibits a higher *G*′ within the LVR compared to the LM ink. This suggests that the incorporation of nickel particles and ultrasonic treatment improves the ink’s ability to resist gravitational deformation. As the strain increases beyond the critical value, both moduli decrease, eventually converging to a crossover point (*G*′ = *G*″) corresponding to the flow point, which also determines the yield stress of the ink. The NLM and pure LM inks exhibit yield stresses of 130 and 117 Pa, respectively. These values are appropriate for DIW printing, ensuring that the inks can be extruded under reasonable pressure while possessing sufficient stress resistance to prevent immediate flow after deposition. Beyond this flow point, *G*″ surpasses *G*′, marking the transition from a solid-like gel to a liquid-like sol, which is a prerequisite for continuous flow through the nozzle.

To evaluate the thixotropy and structural recovery of the LM-based inks, 3-interval thixotropy tests (3ITTs) were conducted by alternating the shear rate. As shown in Fig. 2J, the viscosity of pure LM decreases dramatically from ~1.4 × 10^4^ to ~3.8 Pa·s as the shear rate increases from 0.01 to 30 s^−1^, a behavior primarily attributed to the rupture of the Ga₂O₃ oxide layer [44]. With the incorporation of nickel particles and ultrasonic treatment, the initial viscosity of the NLM ink increases to ~2.3 × 10^4^ Pa·s. Crucially, the NLM ink maintains distinct shear-thinning behavior. At a high shear rate (30 s^−1^), its viscosity drops sharply to ~6.5 Pa·s, yet it rapidly recovers to its initial high value upon reverting to a low shear rate (0.01 s^−1^). After 4 consecutive cycles of alternating shear rates, the viscosity profile remains nearly identical to that of the initial cycle, demonstrating excellent reversibility and structural recovery. 3ITT experiments on the storage and loss moduli also yield consistent findings, as shown in Fig. S5. These results ensure smooth extrusion and excellent self-supporting capability of the ink, thereby enhancing the dimensional accuracy and shape fidelity of the printed structures.

To gain deeper insights into the viscoelastic nature of the inks, frequency sweep tests were conducted to analyze the moduli. As shown in Fig. 2K, the *G*′ of NLM ink is greater than the *G*″ and maintains a stable trend within the low-frequency range (0.01 to 6.3 Hz). This indicates that the NLM ink exhibits solid-like rheological properties under low-shear conditions. At higher frequency (>6.3 Hz), a slight increase in *G*′ is observed. This frequency dependence reflects the structural rigidity of the ink at short timescales, which is beneficial for maintaining shape definition against rapid external disturbances. In summary, this viscoelastic behavior of NLM facilitates precise filament formation while preventing structural collapse during continuous layer stacking [45].

Finally, to evaluate the long-term printing stability of the NLM inks, the modulus variation was monitored over 30 min. As illustrated in Fig. 2L, both *G*′ and *G*″ remain relatively stable throughout the observation period at room temperature. This stability indicates that the ink maintains consistent rheological properties for a sufficiently long duration to span the entire printing process.

### Printing parameters

**Fig. 3.**
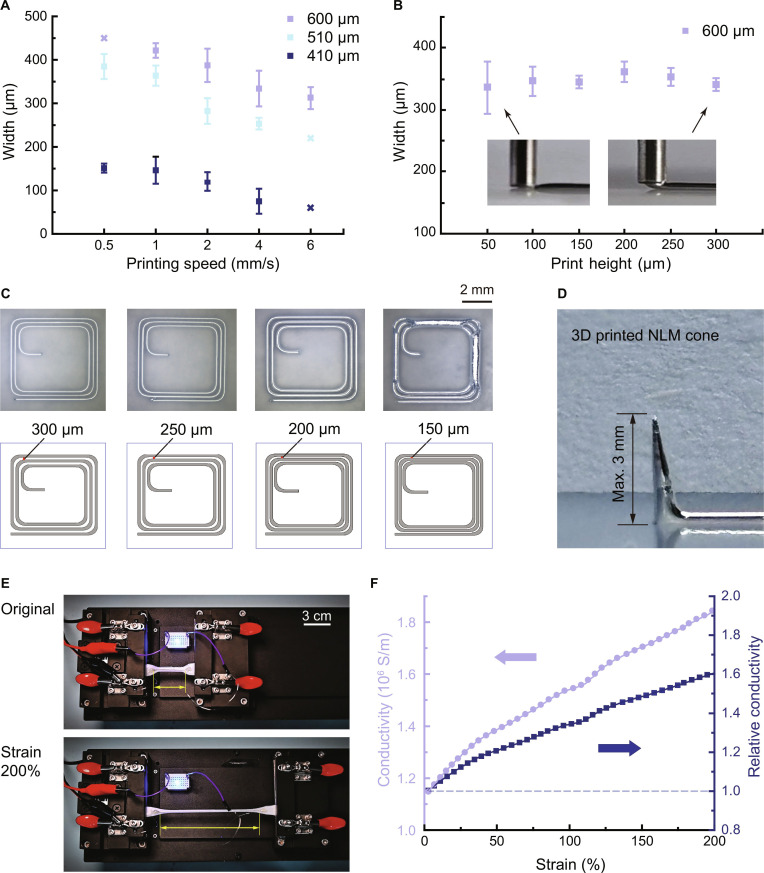
Printing parameters and electromechanical performance of the NLM-elastomer structure. (A) Linewidth dependence on printing speed for nozzle diameters of 600, 510, and 410 μm. (B) Line width versus printing height for a 600-μm nozzle. (C) Printed NLM coils with line spacings from 150 to 300 μm, showing a minimum feasible spacing of 200 μm. (D) Vertically printed NLM cone with a maximum height of 3 mm. (E) Stretchability test of a printed NLM wire encapsulated in a dog-bone elastomer. (F) Absolute and relative electrical conductivity of NLM wire under tensile strain.

The influence of printing speed on the width of printed NLM lines is systematically investigated for nozzles with diameters of 600, 510, and 410 μm under a constant air pressure of 1.5 kPa (Fig. 3A). As the printing speed rises, the width of the printed lines decreases for all nozzle diameters. For the 600-μm-diameter nozzles, the feasible printing speed range is from 1 to 6 mm/s, with a maximum printable line width of 420 μm. At printing speeds ≤0.5 mm/s, LM accumulates and agglomerates, leading to poor printing quality. For the 510- and 410-μm-diameter nozzles, the optimal printing speed range is 0.5 to 4 mm/s. When the printing speed is increased to 6 mm/s, the NLM lines exhibit disconnection, which may result in electrical nonconductivity. Notably, the 410-μm-diameter nozzle enables the fabrication of the narrowest NLM lines, achieving a minimum printable linewidth of 75 μm at a printing speed of 4 mm/s.

To explore the effect of print height on linewidth, experiments are conducted over a printing height range of 50 to 300 μm using a 600-μm-diameter nozzle, under a constant air pressure of 1.5 kPa. The width of the printed line remains relatively stable across this range (Fig. 3B), which suggests that printing height has a negligible impact on linewidth. This stability allows NLM to maintain a nearly uniform linewidth when printed on nonperfectly flat surfaces, specifically the surfaces of printed elastomer layers. This behavior may be attributed to the high surface tension of the LM, which helps the printed lines maintain a consistent shape.

We printed NLM coils with line spacings of 300, 250, 200, and 150 μm (Fig. 3C) to find the minimum printable spacing of the printer. Among these groups, the first 3 groups (300 to 200 μm) show well-defined, continuous patterns. In contrast, when the line spacing is reduced to 150 μm, LM agglomeration occurs, resulting in printing failure. Consequently, the achievable minimum line spacing is 200 μm.

Figure 3D displays a 3D-printed NLM cone, with a maximum height of 3 mm. NLM wire can be printed continuously as long as sufficient ink is supplied and printing pressure is maintained, as shown in Fig. S6A. However, excessive printing height will cause the NLM to agglomerate into spherical lumps or collapse (Fig. S6B). Therefore, the maximum height of the cone is constrained by the inherent rheological properties of the material itself. Extensive experimental results demonstrate that the NLM cone can maintain a stable upright posture when the vertical printing height is ≤3 mm. Figure S6C and D present the NLM cones with vertical printing heights of 2 and 3 mm, respectively, exhibiting stable printing quality.

### Characterization of NLM-elastomer structures

To evaluate the practical functionality of the NLM-elastomer structure, tensile tests combined with electrical conductivity measurements were performed. In Fig. 3E, a blue light-emitting diode (LED) connected in series with the NLM wire serves as a visual indicator of electrical conduction during deformation. The gauge length of the sample is highlighted in yellow for clarity. At the NLM wire’s original length, the LED illuminates upon powering. As the NLM wire is stretched from its original length to 200% strain at a constant speed of 2 mm/s, the LED remains on throughout the entire process (Movie S2). This confirms that the NLM wire embedded within the elastomer maintains electrical conductivity even under strains up to 200%.

The electrical conductivities of the NLM wires are presented in Fig. 3F: the lavender dataset represents absolute conductivity, and the blue curve denotes relative conductivity. Across the strain range, 0% to 200%, both curves exhibit a steady monotonic increase, even as the NLM cross-sectional area of wires decreases. This trend is attributed to the enhanced interaction between the metal-encapsulated regions during the stretching and compression process [32]. Quantitatively, the absolute conductivity rises from 1.15 × 10^6^ S/m at 0% strain to 1.85 × 10^6^ S/m at 200% strain, while relative conductivity climbs from 1.0 to 1.6 over the same interval.

To further verify the excellent reliability and durability of our 3D printing strategy, a cyclic actuation test was conducted on a SEMA consisting of 2 NLM layers. The actuator was driven by a 7-Hz square wave current with an amplitude range of 0.15 to 0 A in a 1-T static magnetic field generated by a vertical electromagnet. As shown in Movie S3, the SEMA maintains normal operation after 200,000 bending cycles (lasting more than 8 h), which validates the superior reliability of our 3D printing strategy.

These results confirm the exceptional electromechanical performance of the NLM-elastomer composite structure. The inherent fluidity and stretchability of NLM enable it to maintain reliable conductivity even under large deformation. Furthermore, the integrated multilayer structure exhibits superior durability.

### 3D-printed SEMA with multilayer coils

**Fig. 4.**
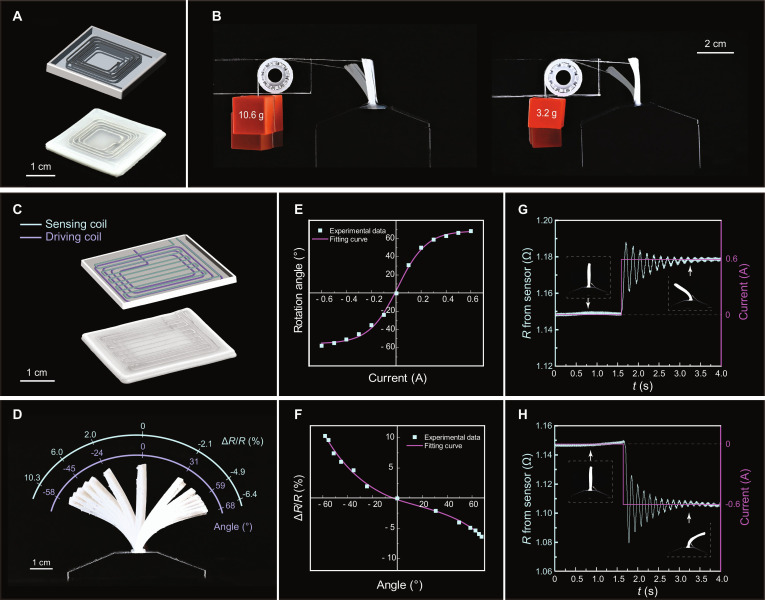
Characterization of 3D-printed SEMAs. (A) A 4-layer-coil SEMA. (B) Experimental setup used to evaluate the output force of a 4-layer-coil and a single-layer-coil SEMAs. (C) A self-sensing SEMA incorporating driving and sensing LM coils. (D) Side-view images of the self-sensing SEMA under a square wave current ±0.6 A, showing rotation angles with corresponding relative resistance changes of the sensing LM coils. (E) Rotation angle as a function of the applied current, with experimental data points and fitted curve. (F) Correlation between relative resistance change and rotation angle. (G and H) Real-time resistance response of the sensing layer during actuation triggered by a step current from 0 to ±0.6 A.

A square SEMA with 4 layers of LM coils was fabricated (Fig. 4A) to demonstrate the advantages of SEMAs with multilayer coils. For comparative analysis, a same-sized SEMA featuring a single-layer coil was used as a control group. The experimental setup for evaluating force output is illustrated in Fig. 4B: the bottom edges of these 2 SEMAs are fixed to the bases, and their top sides are connected to weights via fixed pulleys (Movie S4). Both groups are then placed in a 1-T magnetic field. In the initial state (without current), both actuators are pulled into a bent shape by the attached weights. Upon applying a 1-A current through the coils, the 4-layer-coil SEMA can lift a 10.6-g weight and maintain a stable balanced state in a vertical position. At this point, the torque generated by the Laplace force is in equilibrium with the external torque, as schematically illustrated in Fig. S7. In contrast, the single-layer-coil SEMA can only pull up a 3.2-g weight (details in the Supplementary Text). It is evident that compared with the single-layer-coil SEMA, the 4-layer-coil SEMA exhibits considerably higher external force output. Furthermore, we calculate the volumetric force density $f_{v}=F/\left(L^{2}t\right)$, where *F* is the generated force, *L* = 30 mm is the side length, and *t* is the thickness of the actuator. With thicknesses of 1.45 and 2.89 mm for the single- and 4-layer-coil SEMAs, the corresponding force densities are obtained as 2.41 × 10^4^ and 4.00 × 10^4^ N·m^−3^, respectively. The 4-layer-coil SEMAs therefore achieve approximately 66% higher volumetric force density despite their larger thickness. This clearly highlights that multilayer integration not only boosts absolute force output but also improves force efficiency per unit volume, demonstrating the superior actuation capability of multilayer coil SEMAs and enabling them to meet the requirements of applications that demand higher actuation forces.

### Self-sensing SEMA with integrated multilayer coils

Leveraging the flexibility of 3D printing technology and multilayer printing techniques, a self-sensing SEMA was fabricated. This SEMA comprises 2 coil layers: the first serves as the sensing layer, and the second serves as the driving layer (Fig. 4C). The bottom end of the SEMA is fixed to a base and the entire setup is placed in a 1-T magnetic field (Fig. 4D). When a current ranging from −0.6 to 0.6 A is applied to the driving layer, the SEMA bends at different angles under the action of the Laplace force (Fig. 4E). Specifically, at 0.6 A, it bends rightward by up to 68°, while at −0.6 A, it bends leftward by up to 58°. The numerical difference in bending angles between the 2 directions arises from structural asymmetry of the printed SEMA, primarily due to the nonuniform distribution of EPPC and ECO elastomers. Meanwhile, by detecting changes in the resistivity of the sensing layer, a one-to-one correspondence between resistivity and bending angle can be established (Fig. 4F). This monotonic relationship arises because the sensing-layer LM coil deviates from the neutral axis, experiencing tensile strain in one bending direction and compressive strain in the opposite direction. Thus, positional information can be obtained from resistivity data even when the driving current data are unavailable.

To further analyze the rapid responsiveness of the self-sensing SEMA, Fig. 4G and H illustrate the resistance dynamics of the sensing layer during actuation. In both cases, upon suddenly applying a current from 0 to +0.6 A and −0.6 A, respectively, to the driving layer, the sensing layer’s resistance first undergoes rapid changes, followed by short-lived oscillations, and finally stabilizes at a steady state (Movie S5). For quantification, the response time is defined as the duration from the onset of the resistance change to when the resistance settles within 0.3% of its respective final steady-state value. The resistance data indicate that all response times remain within 1.5 s. This rapid responsiveness reflects the SEMA’s strong mechano-electrical coupling, which validates that it can achieve nearly real-time position detection during actuation.

### Sensor-integrated soft gripper

**Fig. 5.**
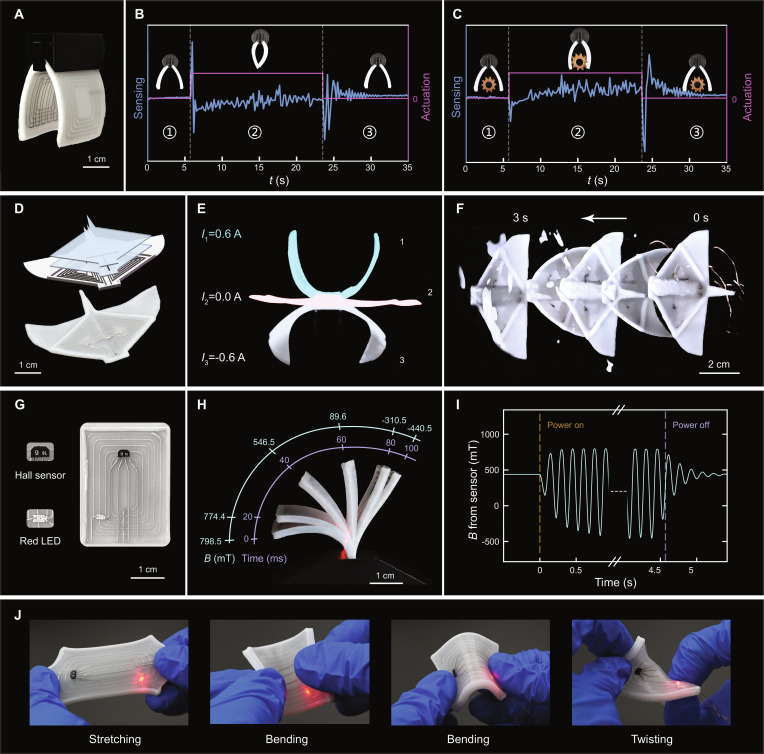
Characterization of 3D-printed SEMRs and a soft SEMA with electronics. (A) A sensor-integrated soft gripper. (B and C) Resistance signals of the soft gripper in grasping experiments without and with a load. (D) Bioinspired manta-like SEMR with double-layer coil architecture. (E) Snapshots of flapping motion in air under ±0.6 A. We name the postures of the SEMR as 1, 2, and 3, indicated on the right side. (F) Swimming performance of the manta-like SEMR in an aqueous environment. (G) A 3D-printed SEMA integrated with electronics: a Hall sensor and a red LED. (H) Magnetic field strength vertically passing through the Hall sensor at different positions during the device’s single swing. (I) Variation in magnetic field strength detected by the Hall sensor when the actuator bends left and right periodically. (J) Robustness test of the 3D-printed SEMA integrated with working electronics: a Hall sensor and a red LED.

Based on the design principle of the self-sensing SEMA, we developed a sensor-integrated soft gripper, which is composed of 2 SEMAs, where the left SEMA was integrated with a resistive sensing module (Fig. 5A). The entire structure of the soft gripper was fabricated using our multimaterial 3D printing strategy.

To evaluate the gripper’s gripping and sensing functions, we performed grasping experiments with and without a load, as shown in Movie S6. The gripper is fixed to a 3D moving platform and placed in a static magnetic field with an amplitude of 1 T. Figure 5B and C show the resistance of the sensing module during a complete actuation of the gripper. The actuation can be categorized into 3 periods. In period 1, the gripper is in its natural, unactuated state, where the resistance is almost stable. In period 2, upon applying a 1-A current, the gripper closes immediately because of the Laplace force, inducing a pronounced transient spike in the sensing signal. Subsequently, the gripper is guided over a prescribed trace together with the 3D moving platform controlled by G-code. Without a load, the sensor resistance in period 2 remains generally lower than that in period 1, whereas it increases in the object-loaded condition. These 2 actuations (Fig. 5B and C) exhibit a clearly distinguishable resistance signal, demonstrating that the gripper not only performs reliable grasping but also possesses excellent self-sensing capability. The minor fluctuations observed during this period primarily arise from slight wobbling and elastic deformation of the soft gripper as it moves. In period 3, once the gripper reaches the targeted position, the driving current is turned off. The measured resistance undergoes another sharp spike, indicating considerable wobbling of the SEMA after the force is released. After approximately 5 s, the gripper stabilizes at its natural position. Although the gripper is in a free state in both period 1 and period 3, the resistance in period 3 is slightly higher than that in period 1. This increase is attributed to the heating effect of the electric current, which raises the SEMA’s temperature and, consequently, its resistance. However, such an effect can be greatly reduced by designing a normally closed gripper with which the current is only applied to open the gripper in a short time to grip or release the object. Such a design can be readily achieved by replacing the gripper’s fixing structure, as shown in Fig. S8.

The sensor-integrated soft gripper demonstrates the advantage of our multimaterial 3D printing strategy in monolithically fabricating devices that integrate both actuation and sensing functionalities. This capability opens new possibilities for more compact and functionally integrated designs in soft robotic systems.

### Manta-like swimming SEMR

To verify the feasibility of the multimaterial 3D printing strategy and Laplace-force actuation mechanism, a manta-like swimming SEMR with a double-layer coil was fabricated (Fig. 5D), with its actuation principle presented in Fig. 1A. The size of the SEMR is 44 mm in body length and 64 mm in wingspan. When the SEMR is placed in a 1-T horizontal magnetic field in air, it remains flat at a driving current of 0 A. Upon applying driving currents of ±0.6 A, the SEMR reaches 2 limit positions, upper flapping and lower flapping postures, as illustrated in positions 1 and 3 in Fig. 5E, respectively. When immersed in water, the robot achieves an average swimming speed of 29 mm/s, corresponding to 0.66 body lengths per second, under a ±0.6 A square-wave current at 3.3 Hz (Fig. 5F and Movie S7). We added a comparative table clarifying our manta-like SEMR’s performance positioning (Table S4), showing its relatively high swimming speed. This practical example of a soft robotic fish demonstrates that our integrated 3D printing technology is reliable and holds great promise for applications in soft robotic systems.

### 3D-printed SEMA integrated with electronics

Figure 5G presents a 3D-printed SMEA integrated with 2 electronic components: a Hall sensor (sensing unit) and a red LED (visual indicator). Such a SEMA can measure the bending angle of the SEMA via the Hall sensor. This device comprises 3 NLM layers, including 2 driving layers and 1 sensing layer with embedded electronic components (Fig. S9).

In the performance characterization, the SEMA is placed in a 1-T uniform static magnetic field. A 7-Hz square wave current (±0.5 A) is applied to the LM coils in the driving layers. Meanwhile, the Hall sensor detects the magnetic field strength component vertically passing through the SEMA. Owing to the structural and material asymmetry in the design, the SEMA exhibits asymmetric swing amplitudes. As illustrated in Fig. 5H, during the SEMA’s bending from the leftmost to the rightmost position, the detected magnetic field strength varies from 798.5 to −440.5 mT. Figure 5I demonstrates that the SEMA exhibits excellent real-time responsiveness in magnetic field detection. During the periodic swing of the SEMA, the detected magnetic field strength exhibits synchronous periodic fluctuations, which are consistent with the periodic actuation of the driving layers. This synchronization confirms that the integrated sensing system can reliably capture dynamic magnetic signals in real time, satisfying the requirements for dynamic monitoring in soft electronic applications. Mechanical robustness is critical for soft electronics operating under complex deformations. As shown in Fig. 5J, the red LED remains illuminated throughout stretching, repeated bending, and twisting cycles (Movie S8), which demonstrates the sustained electrical conductivity and structural integrity of the device.

Collectively, these results validate that our 3D-printed SEMA integrates reliable sensing and actuation capabilities. Its excellent real-time magnetic sensing performance and superior mechanical robustness demonstrate the feasibility of our 3D printing strategy for fabricating high-performance soft electronics.

## Discussion

The proposed strategy achieves direct 3D printing of NLM and elastomer inks within a single streamlined process, thereby reducing manual intervention and improving reproducibility. In contrast to single-material printing, which yields only structural or conductive components, our method produces fully integrated devices with greater fabrication integrity and no need for postprocessing. Compared to other multimaterial approaches, our strategy further distinguishes itself by ensuring that the printed conductors maintain relatively high electrical conductivity (1.15 × 10^6^ S/m at no strain, increasing to 1.85 × 10^6^ S/m at 200% strain). In addition, it supports the reliable fabrication of complex coil architectures, with a minimum line width of 75 μm, a minimum line spacing of 200 μm, and stable vertical NLM connections up to 3 mm in height. Overall, the multimaterial 3D printing strategy is a reliable and generalizable approach for fabricating SEMRs and soft electronics.

In this work, we demonstrate that the proposed multimaterial 3D printing strategy enables reliable fabrication of soft robots and electronics with multilayer coil architectures and integrated funcsachieves a force density 66% higher than that of the single-layer SEMA, resulting in superior actuation performance. Moreover, the integration of sensing and actuation modules within a one-step printing sequence highlights the method’s advanced integrated fabrication capability. Experimentally, the sensing layer can track the deformation and posture of the SEMA within milliseconds, opening the possibility for closed-loop feedback control for soft robots. Based on the self-sensing SEMA, we fabricated a sensor-integrated soft gripper that not only performs reliable grasping but also exhibits excellent self-sensing capability. The successful deployment of this method in a manta-inspired SEMR, which achieves a swimming speed of 29 mm/s, further verifies its effectiveness in motion-intensive application scenarios. Additionally, a SEMA integrated with electronics, a Hall sensor and a red LED, with excellent mechanical robustness. Collectively, these demonstrations highlight the distinct benefits of our 3D printing strategy in achieving streamlined fabrication of high-performance soft robots and electronics with multilayer coil architectures and integrated functionalities.

While this method is particularly effective for vertically continuous architectures, its applicability to hollow structures remains limited by the layer-by-layer deposition scheme. This limitation could be addressed by introducing suitable support materials during printing to sustain the hollow regions, which can then be removed once the overall structure is completed.

This multimaterial 3D printing strategy enables integrated fabrication of functional components, facilitates powerful actuation, and supports miniaturized fabrication, making it highly suitable for biomedical soft robots. In the future, we aim to extend this technique to the fabrication of microscale medical soft electromagnetic robotic collectives.

## Materials and Methods

### Materials

The preparation of EPPC requires Ecoflex 00-30 (Smooth-On, Inc., USA), PDMS (Sylgard 184, Dow Corning, USA) solution, and PTFE powders (Yixin Suhua Co., Ltd., China). The LM composed of gallium (Ga), indium (In), and tin (Sn) with a mass ratio of 68.5:21.5:10 was purchased from Huatai Co., Ltd. (China). The mass density and electrical conductivity are approximately 6.44 g/cm^3^ and 3.46 × 10^6^ S/m at room temperature. Nickel powder with particle diameters ranging from 5 to 8 μm was supplied by Tuopu Metal Materials Co., Ltd.

### Preparation of the printing inks

Three distinct inks are required for 3D printing of multilayer SEMAs. The first ink is EPPC, formulated by mixing ECO, PDMS solution, and PTFE powders at a mass ratio of 3:2:5. The ECO solution is prepared by mixing Part A and Part B at a 1:1 mass ratio, while the PDMS solution consists of PDMS base and curing agent with a mass ratio of 10:1. These 3 components are blended and degassed in a planetary mixer (BHZ-300, Beihong Co., Ltd.) under vacuum conditions (−98 kPa), through a 2-step process for 1 min at 0 rpm and 6 min at 1,000 rpm. The mixed solution retains its printability for up to 2 h at room temperature and up to 24 h when stored at −18 °C.

The second type is the NLM ink, which is used to print conductive coils in SEMAs. This ink is formulated by adding nickel microparticles at 8 wt% of the LM mass to the LM. Initially, the nickel powder floats on the surface of the LM. After 24 min of stirring (at 1,000 rpm), all particles are encapsulated within the LM matrix. To ensure homogeneous dispersion, high-shear mixing is subsequently performed for 7 min using an ultrasonic cell disruptor (Lichen Co., Ltd., China) equipped with a 6-mm titanium-coated amplitude transformer. Ultrasonication is conducted at an ultrasonic power of 300 W and a sonic frequency of 25 kHz, using a pulse mode, 2 s on and 4 s off.

The third one is the ECO solution, which is obtained by mixing Part A and Part B at a 1:1 mass ratio. After blending, the mixture is vacuumed at −80 kPa for 3 min to eliminate air bubbles, yielding a printable ink.

### 3D printing system

The multimaterial printing system is primarily composed of a high-resolution 3D motion stage (Qiyu Technology Co., Ltd.), a heated bed, replaceable syringes, a pressure dispenser (Ultimus V, Nordson Inc.), and a personal computer for system control (Fig. S10). The syringes loaded with EPPC, NLM, and ECO inks are connected to the pressure dispenser, which enables precise regulation of the pressure within the nozzle.

### Manufacturing procedure of SEMAs

Structures of SEMAs are designed and constructed using SolidWorks (Dassault Systèmes) and then sliced into G-code files for the 3D printer. For the multimaterial printing process, the syringes containing the respective inks are manually mounted on the moving carriage. For material switching, the syringe is replaced, after which the printer resumes deposition by executing the corresponding G-code file. The printing sequence is illustrated in Fig. 1D. In the curing process, a 375-W infrared heating lamp (Zhenzhen Optoelectronics Co., Ltd.) is employed, with a wavelength range of 760 to 5,000 nm. Printing parameters for each printing material, including nozzle type, nozzle diameter, printing speed, and applied air pressure, are provided in Table S2. In addition, to form the interlayer connecting cone, a vacuum pressure of 3 kPa is applied upon finishing cone printing. The NLM wire spontaneously disconnects at the lower end of the needle tip, thus achieving the designed height.

### Roughness characterization

To evaluate the surface roughness of the printed elastomer layers, a high-precision laser displacement sensor (STM250, Shenzhen Suoertai Sensor Co., Ltd.) is mounted onto the 3D motion stage. The sensor movement is automated via a custom G-code program. The scanning paths are oriented perpendicular to the printing trajectories to effectively capture the maximum topological fluctuations across the deposited filaments.

### Mechanical characterization

For the tensile tests, 4 groups of dumbbell-shaped specimens are fabricated, namely, printed EPPC, molded EPPC, printed ECO, and molded ECO. All specimens are designed to conform to the dimensional specifications of ISO 37:2005 Type 2. For the T-peel tests, 3 groups of rectangular specimens with dimensions of 60 mm × 20 mm are prepared. Each specimen consists of 2 layers that are cured separately. Prior to printing the second layer, an adhesive tape with dimensions of 30 mm × 20 mm is affixed to one end of the first layer to isolate the 2 layers, while the opposite end is allowed to cure naturally without any physical separation. After the second layer is also cured, the adhesive tape is removed, and the specimens for the peel test are obtained. Since liquid-state ECO ink exhibits low shape fidelity, a polylactic acid frame is used to confine the ink during the DIW printing process. These samples are tested on a tensile testing machine (Instron 5465, Norwood, USA) equipped with a 1-kN force sensor at a tensile rate of 20 mm/min to characterize their mechanical properties. Young’s moduli are calculated as the average of 5 samples per group, derived from the slopes of the stress–strain curves within the initial 30% strain region.

### Rheological characterization

Rheological measurements are performed using a rotational rheometer (Discovery HR-20, TA Instruments, USA) equipped with a 20-mm parallel plate geometry and a fixed 1-mm gap. The variation of ink viscosity with increasing shear rate is measured under flow-controlled mode at 25 °C. For elastomer-based inks, the curing behavior is recorded at 60 °C under a constant strain of 0.9% and an angular frequency of 1.6 Hz. For LM-based inks, frequency sweep experiments are conducted over a frequency range of 0.01 to 100 Hz at a strain of 0.9% and 25 °C to assess viscoelastic behavior. The amplitude sweep experiments are carried out at a strain range of 0.1% to 100% at a frequency of 1 Hz and 25 °C to determine the LVR and yield stress. Additionally, the time-dependent evolution of storage modulus (*G*′) and loss modulus (*G*″) is monitored for a 30-min duration under constant conditions (0.9% strain, 1.6 Hz, 25 °C). Thixotropic properties are characterized via the 3ITTs in 2 modes. Rotational tests alternate shear rates between 0.01 s^−1^ (within the LVR) and 30 s^−1^(destructive state), while oscillatory tests (at 1 Hz) alternate strains between 0.16% (LVR) and 477% (destructive state). Each experiment is performed at least 3 times, demonstrating good repeatability. One set of the results is selected for presentation.

### Electrical property measurements

The resistance of the NLM wire embedded in the elastomer is measured using the 4-wire method with a 7-digit digital multimeter (34470A, Keysight). First, the 2 ends of the NLM wire are extended using silver wires. Then, attach the test leads of the multimeter to the ends of the silver wires. The resistance of the NLM wire is calculated as the total resistance minus the resistance of the 2 silver wires. The length and cross-sectional area of the NLM wire are determined from optical microscope images (AO-HK830-0318, Aosvi, China) and quantified with ImageJ software (NIH, USA). The electrical conductivity is then calculated by averaging the measurements from 3 samples.

In the case of the self-sensing SEMA, the resistance of the sensing layer is measured by constructing a constant-voltage source circuit. Specifically, the sensing layer coil is connected in series with a high-value resistor. The coil voltage is recorded in real time using a voltage acquisition card (USB8865, Art Technology Co., Ltd.), which enables fast and continuous monitoring. The resistance is then derived indirectly from the acquired voltage data.

### Characterization of the electromagnets

Two electromagnets are employed in the experimental setup: one vertical and one horizontal. These 2 electromagnets (Model PEM-2014, supplied by Litian Magnetoelectric Technology Co., Ltd.) share identical specifications except for their orientation, featuring a pole face diameter of 200 mm, an operating air gap of 80 mm, and operating at 24 A with 300 V. The magnetic fields generated by the electromagnets are characterized using a Gauss meter (Model 931, Honor Top Magnetic Technology Co., Ltd.). As shown in Figs. S11 and S12, field measurements are taken at 33 points on the planes located at *Z* = 10, 40, and 70 mm. The magnetic field distribution is reconstructed in MATLAB using data obtained by averaging 3 sets of measured data. Specifically, the sampling range is first mapped onto a 500 × 500 regular grid, followed by cubic interpolation. This interpolation method constructs cubic polynomials between data points, thereby producing smooth and continuous results across the interpolated grid. The interpolated fields provide an intuitive visualization of the magnetic field distribution characteristics.

### Control and power system of the manta-like SEMR

The control system consists of an Arduino Uno board and 2 L298N H-bridge driver modules (Fig. S13). The control algorithm, written in the Arduino Integrated Development Environment (IDE), is deployed and executed on the Uno board. The Uno board controls the 2 L298N modules to generate 2 square-wave current signals with adjustable frequency and symmetric amplitude (±0.6 A). Two independent DC power supplies (IT6833A, ITECH) provide stable and regulated power to the H-bridge circuits.

### Electronic components embedded in elastomers

The Hall sensor adopts the Toshiba THS119 with a SOT-23 package. The red LED is a surface-mount device with a 1206 package from Shenzhen Feiteng Microelectronics Firm. After printing the NLM wires, the electronic components are placed at the designed positions using tweezers, and finally covered with a layer of ECO to encapsulate all components and wires.

## Data Availability

All data needed to evaluate the conclusions of the paper are available in the paper or the Supplementary Materials. Additional data related to this paper may be requested from the authors.

## References

[1] Yue T, Lu C, Tang K, Qi Q, Lu Z, Lee LY, Bloomfield-Gadȇlha H, Rossiter J. Embodying soft robots with octopus-inspired hierarchical suction intelligence. Sci Robot. 2025;10(102):eadr4264. 40367198 10.1126/scirobotics.adr4264

[2] Del Dottore E, Mondini A, Rowe N, Mazzolai B. A growing soft robot with climbing plant–inspired adaptive behaviors for navigation in unstructured environments. Sci Robot. 2024;9(86):eadi5908. 38232147 10.1126/scirobotics.adi5908

[3] Li G, Shen P, Wong T-W, Liu M, Sun Z, Liu X, Chen Y, Wang X, Zhang H, Hu B, et al. Plasticized electrohydraulic robot autopilots in the deep sea. Sci Robot. 2025;10(105):eadt8054. 40802732 10.1126/scirobotics.adt8054

[4] Sankar S, Cheng W-Y, Zhang J, Slepyan A, Iskarous MM, Greene RJ, DeBrabander R, Chen J, Gupta A, Thakor NV. A natural biomimetic prosthetic hand with neuromorphic tactile sensing for precise and compliant grasping. Sci Adv. 2025;11(10): Article eadr9300. 40043132 10.1126/sciadv.adr9300 PMC11881920

[5] Su J, Tan JMR, Liu J, He K, Wu D, Lai W, Cao J, Phee SJ, Magdassi S, Chen X. Bioarchitectonics-inspired soft grippers with cutaneous slip perception. Sci Adv. 2025;11(33): Article eadr9300. 40802770 10.1126/sciadv.adx4206 PMC12346268

[6] Ching T, Lee JZW, Win SKH, Win LST, Sufiyan D, Lim CPX, Nagaraju N, Toh Y-C, Foong S, Hashimoto M. Crawling, climbing, perching, and flying by FiBa soft robots. Sci Robot. 2024;9(92): Article eadk4533. 39018373 10.1126/scirobotics.adk4533

[7] Wang W, Jiang Y, Zhong D, Zhang Z, Choudhury S, Lai J-C, Gong H, Niu S, Yan X, Zheng Y, et al. Neuromorphic sensorimotor loop embodied by monolithically integrated, low-voltage, soft e-skin. Science. 2023;380(6646):735–742. 37200416 10.1126/science.ade0086

[8] Li C, Cheng J, He Y, He X, Xu Z, Ge Q, Yang C. Polyelectrolyte elastomer-based ionotronic sensors with multi-mode sensing capabilities via multi-material 3D printing. Nat Commun. 2023;14:4853. 37563150 10.1038/s41467-023-40583-5 PMC10415297

[9] Cui Y, Yu W, Li J, Shao Q, Weng D, Yin G, Zhang X, Liu X, Ye J, Wang J, et al. An automatic implementation of oropharyngeal swab sampling for diagnosing respiratory infectious diseases via soft robotic end-effectors. Chin J Mech Eng. 2024;37:29.

[10] Wang Z, Zhang B, He Q, Chen H, Wang J, Yao Y, Zhou N, Cui W. Multimaterial embedded 3D printing of composite reinforced soft actuators. Research. 2023;6: Article 0122. 37223483 10.34133/research.0122 PMC10202188

[11] Kim M, Park JJ, Hong S, Jung Y, Bang J, Cho C, Ko SH. Monolithically stacked VIA-free liquid metal circuit for stretchable electronics. Mater Today. 2025;83:24–34.

[12] Zhou M, Li Z, Wang Z, Li X, Wang R, Li H, Zhang H, Sun W, Wang T, Liu X, et al. All-printed VIA-free polyimide-based multilayer flexible circuits. J Mater Sci Technol. 2026;249:131–141.

[13] Wang Y, Qin H, Liu N, Hu Q-N, Cong H-P, Yu S-H. Robust and fast-transforming soft microrobots driven by low magnetic field. Adv Mater. 2025;37(37):2505193. 10.1002/adma.202505193 40545997

[14] Seong M, Sun K, Kim S, Kwon H, Lee S-W, Veerla SC, Kang DK, Kim J, Kondaveeti S, Tawfik SM, et al. Multifunctional magnetic muscles for soft robotics. Nat Commun. 2024;15(1):7929. 39256389 10.1038/s41467-024-52347-w PMC11387479

[15] Choi Y, Shin G, Yoon SJ, Park Y-L. Soft electromagnetic sliding actuators for highly compliant planar motions using microfluidic conductive coil array. Soft Robot. 2025;12(1):135–144. 39253876 10.1089/soro.2024.0007

[16] Wang C, Wang T, Li M, Zhang R, Ugurlu H, Sitti M. Heterogeneous multiple soft millirobots in three-dimensional lumens. Sci Adv. 2024;10(45):eadq1951. 39504364 10.1126/sciadv.adq1951 PMC11540014

[17] Min H, Bae D, Jang S, Lee S, Park M, Dayan CB, Choi J, Bak K, Yang Y, Chun S, et al. Stiffness-tunable velvet worm–inspired soft adhesive robot. Sci Adv. 2024;10(47):eadp8260. 39565852 10.1126/sciadv.adp8260 PMC11578180

[18] Xia N, Jin D, Yang Z, Pan C, Su L, Zhang M, Wang X, Xu Z, Guo Z, Pan L, et al. Inverse programming of ferromagnetic domains for 3D curved surfaces of soft materials. Nat Synth. 2025;4:642–654.

[19] Zhang L, Zhao S, Zhou X, Jing X, Zhou Y, Wang Y, Zhu Y, Liu X, Zhao Z, Zhang D, et al. A magnetic-driven multi-motion robot with position/orientation sensing capability. Research. 2023;6: Article 0177. 39882544 10.34133/research.0177 PMC11778601

[20] Mao G, Drack M, Karami-Mosammam M, Wirthl D, Stockinger T, Schwödiauer R, Kaltenbrunner M. Soft electromagnetic actuators. Sci Adv. 2020;6(26):eabc0251. 32637626 10.1126/sciadv.abc0251 PMC7319732

[21] Li W, Chen H, Yi Z, Fang F, Guo X, Wu Z, Gao Q, Shao L, Xu J, Meng G, et al. Self-vectoring electromagnetic soft robots with high operational dimensionality. Nat Commun. 2023;14:182. 36635282 10.1038/s41467-023-35848-y PMC9837125

[22] Bartkowski P, Pawliszak Ł, Chevale SG, Pełka P, Park Y-L. Programmable shape-shifting soft robotic structure using liquid metal electromagnetic actuators. Soft Robot. 2024;11(5):802–811. 38598718 10.1089/soro.2023.0144

[23] Ni X, Luan H, Kim J-T, Rogge SI, Bai Y, Kwak JW, Liu S, Yang DS, Li S, Li S, et al. Soft shape-programmable surfaces by fast electromagnetic actuation of liquid metal networks. Nat Commun. 2022;13:5576. 36151092 10.1038/s41467-022-31092-y PMC9508113

[24] Li N, Zhou Y, Li Y, Li C, Xiang W, Chen X, Zhang P, Zhang Q, Su J, Jin B, et al. Transformable 3D curved high-density liquid metal coils—An integrated unit for general soft actuation, sensing and communication. Nat Commun. 2024;15:7679. 39237505 10.1038/s41467-024-51648-4 PMC11377734

[25] Li N, Zhan F, Guo M, Yuan X, Chen X, Li Y, Zhang G, Wang L, Liu J. Fingertip-inspired spatially anisotropic inductive liquid metal sensors with ultra-wide range, high linearity and exceptional stability. Adv Mater. 2025;37(19):2419524. 10.1002/adma.202419524 40135258

[26] Lee B, Cho H, Moon S, Ko Y, Ryu Y-S, Kim H, Jeong J, Chung S. Omnidirectional printing of elastic conductors for three-dimensional stretchable electronics. Nat Electron. 2023;6:307–318.

[27] Sakorikar T, Mihaliak N, Krisnadi F, Ma J, Kim T, Kong M, Awartani O, Dickey MD. A guide to printed stretchable conductors. Chem Rev. 2024;124(3):860–888. 38291556 10.1021/acs.chemrev.3c00569

[28] Sun P, Zhang J, Zhu X, Li H, Li Y, Yang J, Peng Z, Zhang G, Wang F, Lan H. Directly printed interconnection wires between layers for 3D integrated stretchable electronics. Adv Mater Technol. 2022;7(9):2200302.

[29] Li Z, Li H, Zhu X, Peng Z, Zhang G, Yang J, Wang F, Zhang Y-F, Sun L, Wang R, et al. Directly printed embedded metal mesh for flexible transparent electrode via liquid substrate electric-field-driven jet. Adv Sci. 2022;9(14):2105331. 10.1002/advs.202105331 PMC9108624 35233960

[30] Mao G, Schiller D, Danninger D, Hailegnaw B, Hartmann F, Stockinger T, Drack M, Arnold N, Kaltenbrunner M. Ultrafast small-scale soft electromagnetic robots. Nat Commun. 2022;13(1):4456. 35945209 10.1038/s41467-022-32123-4 PMC9363453

[31] Chen W, Tang Q, Zhong W, Lai M, Shi S, Tan J, Luo Z, Liu X, Ye Z, He R, et al. Directly printable and adhesive liquid metal ink for wearable devices. Adv Funct Mater. 2025;35(1):2411647.

[32] Daalkhaijav U, Yirmibesoglu OD, Walker S, Mengüç Y. Rheological modification of liquid metal for additive manufacturing of stretchable electronics. Adv Mater Technol. 2018;3(4):1700351.

[33] Chung WG, Jang J, Cui G, Lee S, Jeong H, Kang H, Seo H, Kim S, Kim E, Lee J, et al. Liquid-metal-based three-dimensional microelectrode arrays integrated with implantable ultrathin retinal prosthesis for vision restoration. Nat Nanotechnol. 2024;19(5):688–697. 38225357 10.1038/s41565-023-01587-w PMC11106006

[34] Park Y-G, An HS, Kim J-Y, Park J-U. High-resolution, reconfigurable printing of liquid metals with three-dimensional structures. Sci Adv. 2019;5(6):eaaw2844. 31245538 10.1126/sciadv.aaw2844 PMC6588379

[35] Zheng R, Chen Y, Chi H, Qiu H, Xue H, Bai H. 3D printing of a polydimethylsiloxane/polytetrafluoroethylene composite elastomer and its application in a triboelectric nanogenerator. ACS Appl Mater Interfaces. 2020;12(51):57441–57449. 33297670 10.1021/acsami.0c18201

[36] Ge Q, Chen Z, Cheng J, Zhang B, Zhang Y-F, Li H, He X, Yuan C, Liu J, Magdassi S, et al. 3D printing of highly stretchable hydrogel with diverse UV curable polymers. Sci Adv. 2021;7(2):eaba4261. 33523958 10.1126/sciadv.aba4261 PMC7787492

[37] Chen Z, Zhao D, Liu B, Nian G, Li X, Yin J, Qu S, Yang W. 3D printing of multifunctional hydrogels. Adv Funct Mater. 2019;29(20):1900971.

[38] Lin Z, Qiu X, Cai Z, Li J, Zhao Y, Lin X, Zhang J, Hu X, Bai H. High internal phase emulsions gel ink for direct-ink-writing 3D printing of liquid metal. Nat Commun. 2024;15:4806. 38839743 10.1038/s41467-024-48906-w PMC11153652

[39] Zhang Y, Pan C, Liu P, Peng L, Liu Z, Li Y, Wang Q, Wu T, Li Z, Majidi C, et al. Coaxially printed magnetic mechanical electrical hybrid structures with actuation and sensing functionalities. Nat Commun. 2023;14:4428. 37481621 10.1038/s41467-023-40109-z PMC10363174

[40] Huang Q, Bao T, Bai J, Pan G, Zhang Y. Kinematics in a manta-like robot for one-degree-of-freedom intermittent propulsion. Phys Fluids. 2025;37(3): Article 031905.

[41] Saadi MASR, Maguire A, Pottackal NT, Thakur MSH, Ikram MM, Hart AJ, Ajayan PM, Rahman MM. Direct ink writing: A 3D printing technology for diverse materials. Adv Mater. 2022;34(28):2108855. 10.1002/adma.202108855 35246886

[42] Krisnadi F, Sakorikar T, Vong MH, Dickey MD. Improved direct ink writing of liquid metal foams via liquid additives. Adv Electron Mater. 2025;2500009.

[43] Wu Y, Deng Z, Peng Z, Zheng R, Liu S, Xing S, Li J, Huang D, Liu L. A novel strategy for preparing stretchable and reliable biphasic liquid metal. Adv Funct Mater. 2019;29(36):1903840.

[44] Xu Q, Oudalov N, Guo Q, Jaeger HM, Brown E. Effect of oxidation on the mechanical properties of liquid gallium and eutectic gallium-indium. Phys Fluids. 2012;24(6): Article 063101.

[45] Huang B, Xu K, Zhang X, Zhang Y, Long Y-Z, Ge SS, Wei X. 3D printing of self-powered triboelectric intelligent system with enhanced output performance for material identification via machine learning. Chem Eng J. 2025;515: Article 163790.

