# Supplementary material for: Integrated 3D Printing of Liquid Metal and Elastomer for Soft Robots and Electronics
Source: Research (Wash D C). 2026 Mar 6;9:1174. doi: 10.34133/research.1174 (PMC12963645; doi:10.34133/research.1174)
Supplement: Supplementary 1 — Supplementary Text Figs. S1 to S13 Tables S1 to S4 Movies S1 to S8 [file research.1174.f1.zip › supplementary material.docx]

Supplementary Materials for

# Integrated 3D Printing of Liquid Metal and Elastomer for Soft Robots and Electronics

Xiaoyu Song *et al.*

*Corresponding author. Email: guoyongmao@zju.edu.cn.

**This PDF file includes:**

Supplementary Text

Figs. S1 to S13

Tables S1 to S4

Legends for movies S1 to S8

**Other Supplementary Materials for this manuscript include the following:**

Movies S1 to S8

Supplementary Text

**Force estimation of the square-shaped SEMAs**

To demonstrate the advantage of multilayer coils in achieving higher energy density, we conducted a comparative experiment on the external forces exerted by single-layer and four-layer coils with the same dimensions. As shown in Fig. S5, the bottom edge of the SEMA is fixed to a base and placed in a vertical magnetic field of 1 T with a 1 A current applied. According to Laplace’s law, only the horizontal liquid-metal channels are considered. For analysis, the driving plate is simplified as a cantilever beam. When the actuator stays at the vertical equilibrium position, the external torque balances the Laplace torque, and the output force exerted by the driving plate can be calculated. The total moment caused by the Laplace force can be calculated as , where is the label of the horizontal channels and is the distance from the channel to the fixed base. For the single-layer coil, as shown in Fig. S5B, the upper four channels generate forces directed to the right, while the lower channels generate forces directed to the left. The resultant torque is calculated to be 0.001 N·m. According to the external force equation , where denotes the distance from the point of action to the fixed base. The output force of the single-layer coil is calculated to be 0.033 N, which theoretically corresponds to a lifting capacity of 3.4 g. For the four-layer SEMA, using the same calculation method, the net torque is 0.0034 N·m, resulting in an output force of 0.113 N, which corresponds to a lifting capacity of 11.5 g. Experimentally, the single-layer actuator achieves a maximum lifting mass of 3.2 g, while the four-layer actuator reaches 10.6 g, which is substantially higher than that of the single-layer actuator, as shown in Movie S3. The discrepancy between the theoretical and experimental results is likely due to the non-uniform distribution of the magnetic field (a uniform field of 1 T was assumed for simplicity in the theoretical calculation) and the influence of friction during the lifting process. Furthermore, we calculated the volumetric force density; the calculation process is presented in the main text and results section.

**Interfacial adhesion analysis**

To evaluate the adhesion stability of the multilayer structures, T-peel tests were conducted (Fig. S4A). Peel strength and adhesion energy are two core metrics for assessing the bonding performance of multilayer structures. Peel strength refers to the force required to resist peeling per unit width, calculated as , where denotes the steady-state average force measured during the peel test, and represents the width of the specimen. Adhesion energy is defined as the energy consumed to debond a unit area of the interface, with its corresponding formula for T-peel tests expressed as (*1*). In practical tests (Fig. S4B), the EPPC-EPPC and ECO-ECO groups undergo stable peeling behavior, with the calculated peel strengths of and , and adhesion energies of and , respectively. These experimental data verify that these two groups demonstrate favorable adhesion performance. On the other hand, the EPPC-ECO group exhibits crack path deviation during the peeling process. This phenomenon arises when the interfacial energy exceeds the fracture energy of the bulk materials, which strongly verifies the superior bonding performance between EPPC and ECO (*2*).


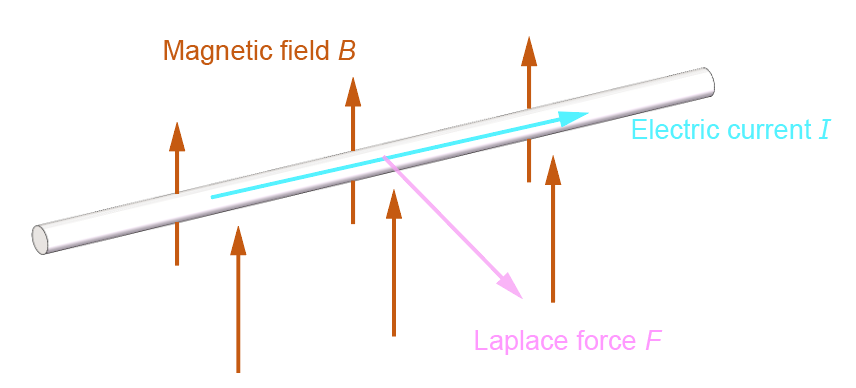


**Fig. S1.** Schematic illustration of the Laplace force on a wire carrying a current in a static magnetic field.


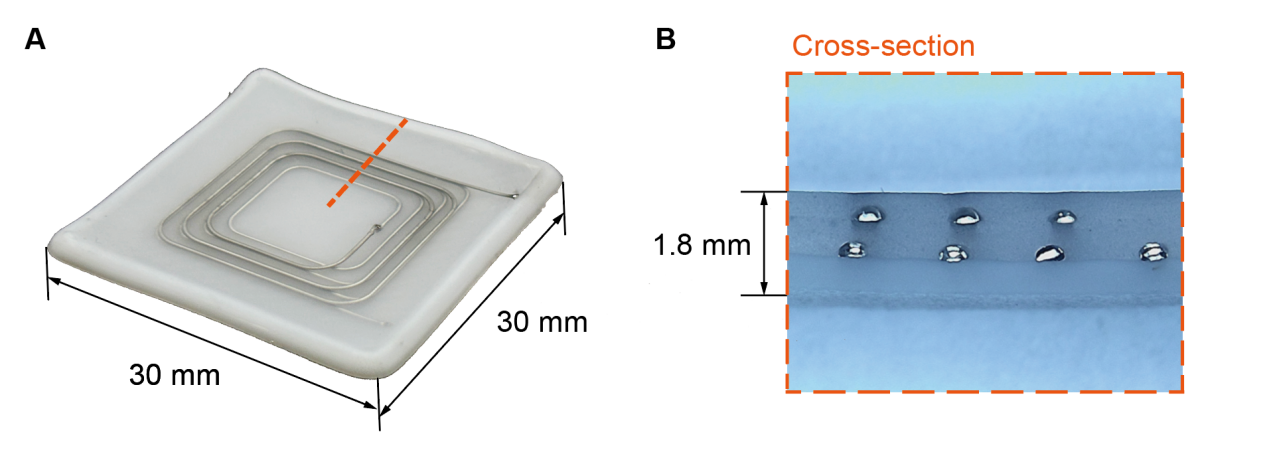


Fig. S2. Structural parameters of the square-shaped SEMA with a double-layer coil. (A) Dimensions of the SEMA. (B) Microscopic picture of the cross-sectional view of the SEMA.

**
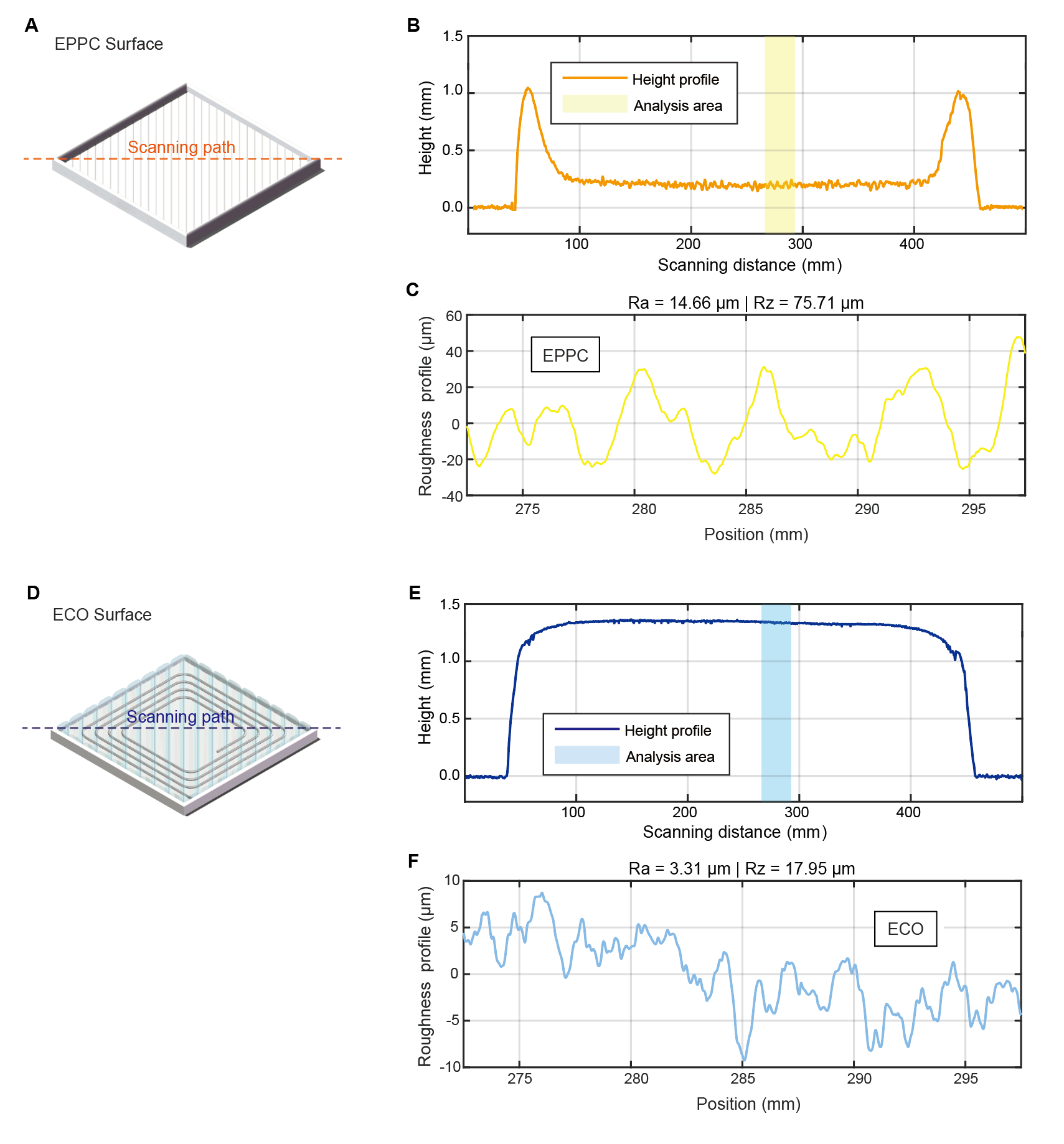
**

**Fig. S3.** Roughness characterization of 3D-printed elastomer layers. (A, D) Scanning path on the (A) EPPC and (D) ECO surface, oriented perpendicular to the printing trajectories. (B, E) Height profile of (B) EPPC and (E) ECO layers. (C, F) Roughness profile analysis within selected 25-mm segments. Compared to the EPPC substrate, which exhibits an arithmetic mean roughness (Ra) of 14.66 μm and a ten-point mean roughness (Rz) of 75.71 μm (C), the ECO layer shows a significantly lower Ra of 3.31 μm and Rz of 17.95 μm (F).


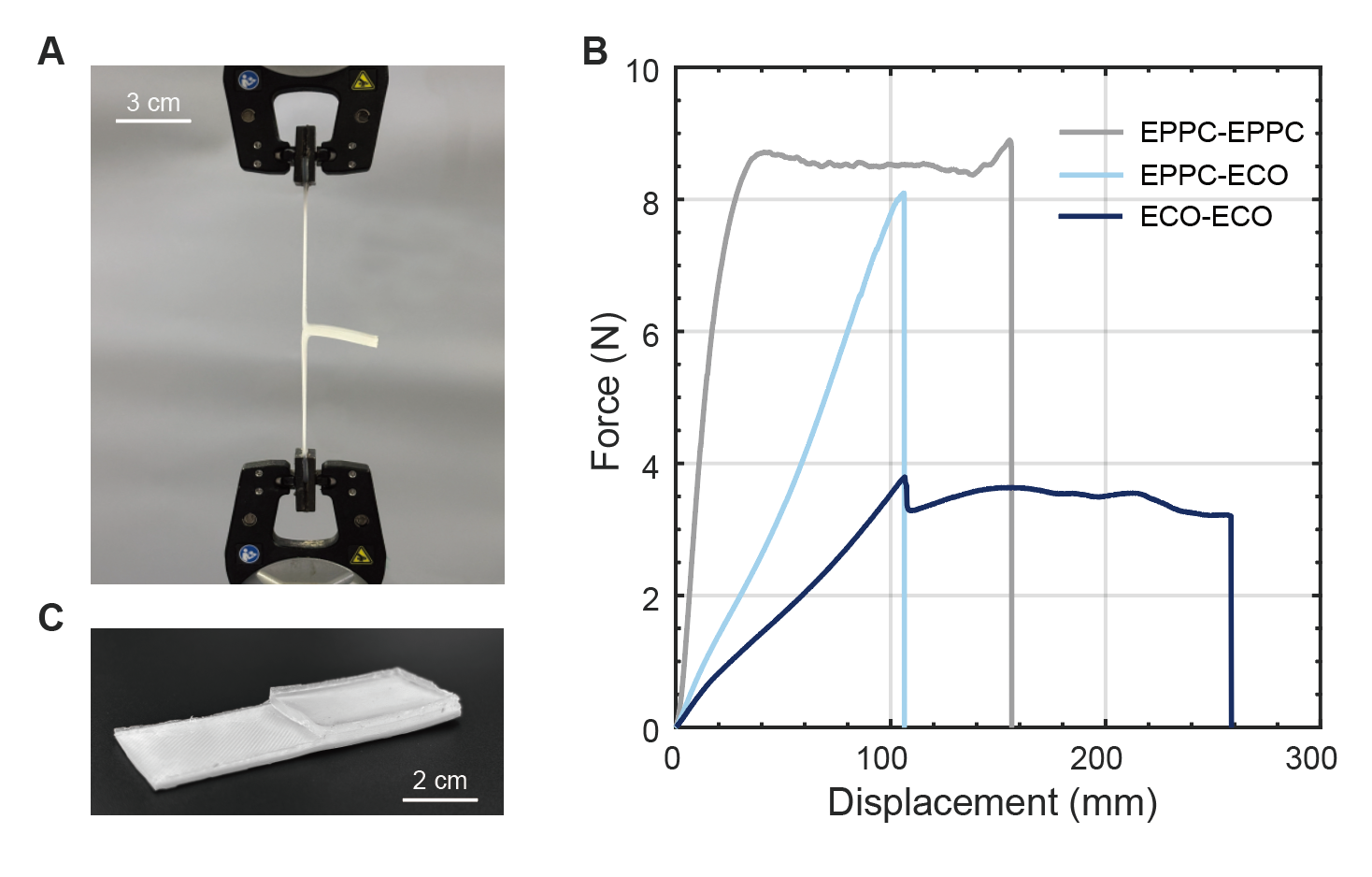


**Fig. S4.** T-peel test. (A) Experimental setup. (B) Force-displacement curves of EPPC-EPPC, EPPC-ECO, and ECO-ECO bonded interfaces. (C) Post-test photograph of the EPPC-ECO bonded sample exhibiting ECO layer fracture.

**
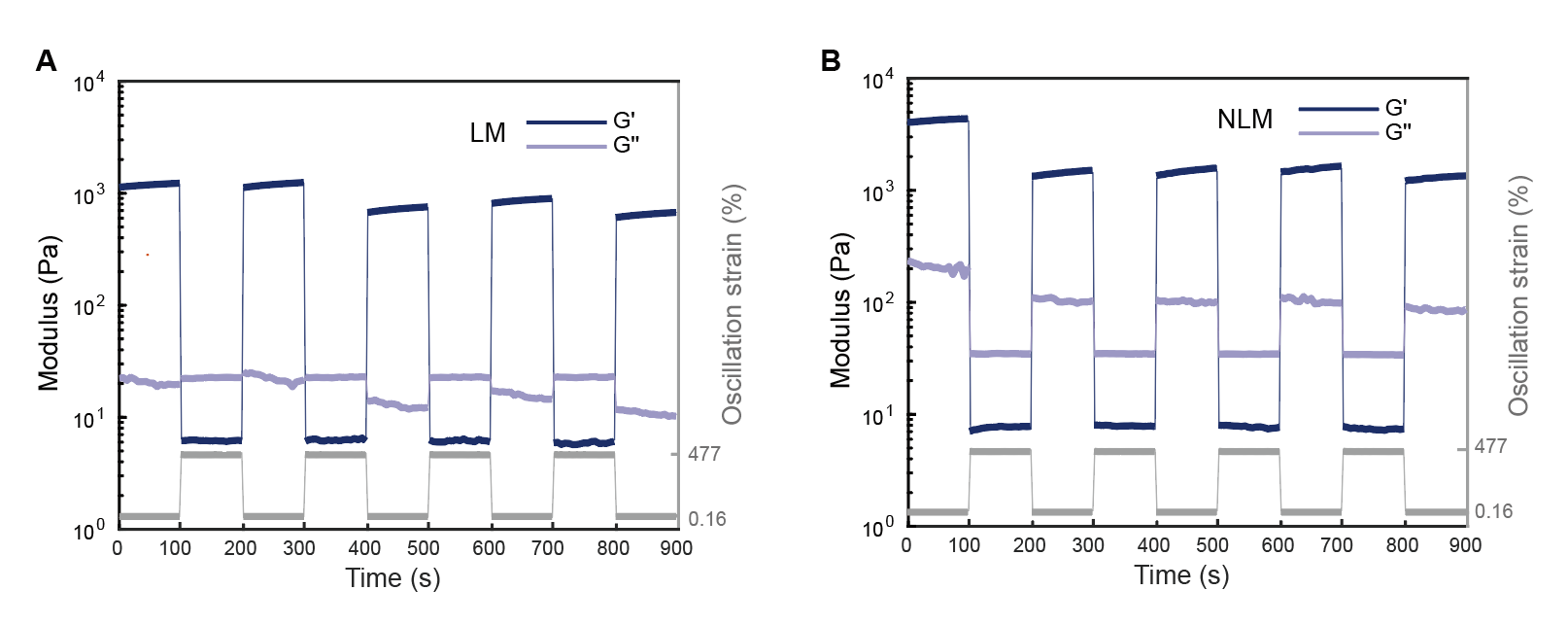
**

**Fig. S5.** Three-interval thixotropy tests for characterizing the modulus variation of (A) LM and (B) NLM.

**
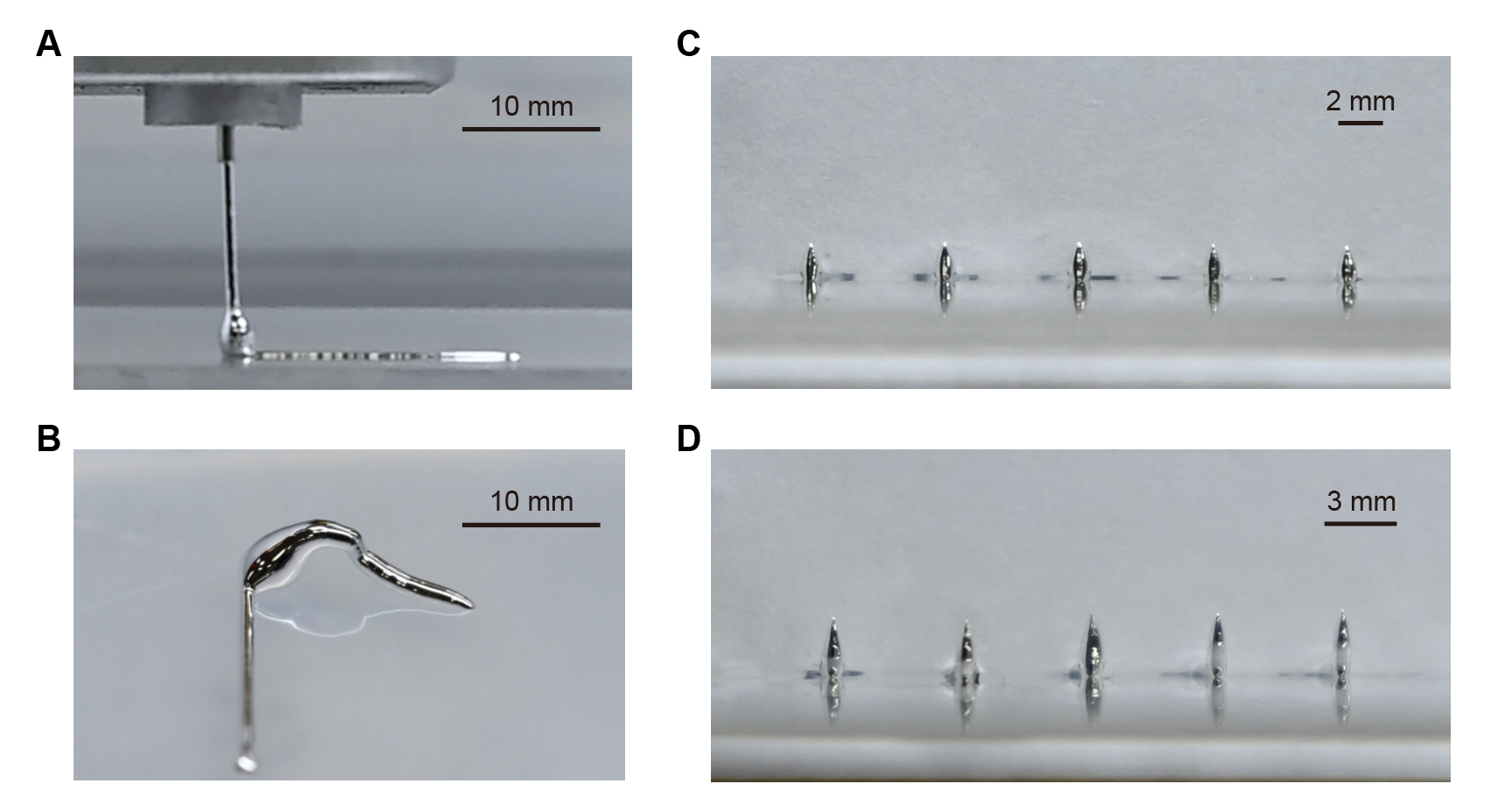
**

**Fig. S6.** Printing height characterization of NLM cones. (A) Continuous vertical printing of NLM wires under sufficient ink supply and stable printing pressure. (B) Agglomeration and structural collapse of NLM induced by excessive vertical printing height. (C) Optical images of NLM cones with a vertical printing height of 2 mm, scale bar: 2 mm. (D) Optical images of NLM cones with a vertical printing height of 3 mm, scale bar: 3 mm.


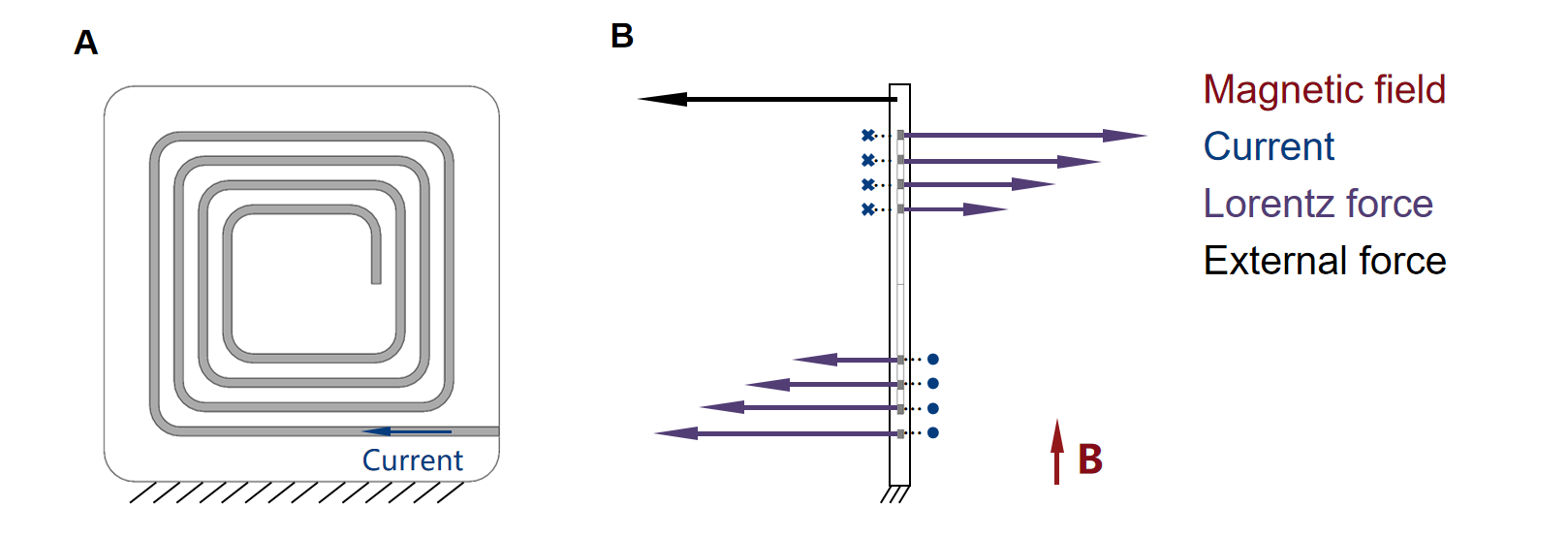


**Fig. S7.** Schematic illustration of the single-layer SEMA under a vertical magnetic field, showing the distributions of the Lorentz force and the external forces. (A) Front view. (B) Left view.


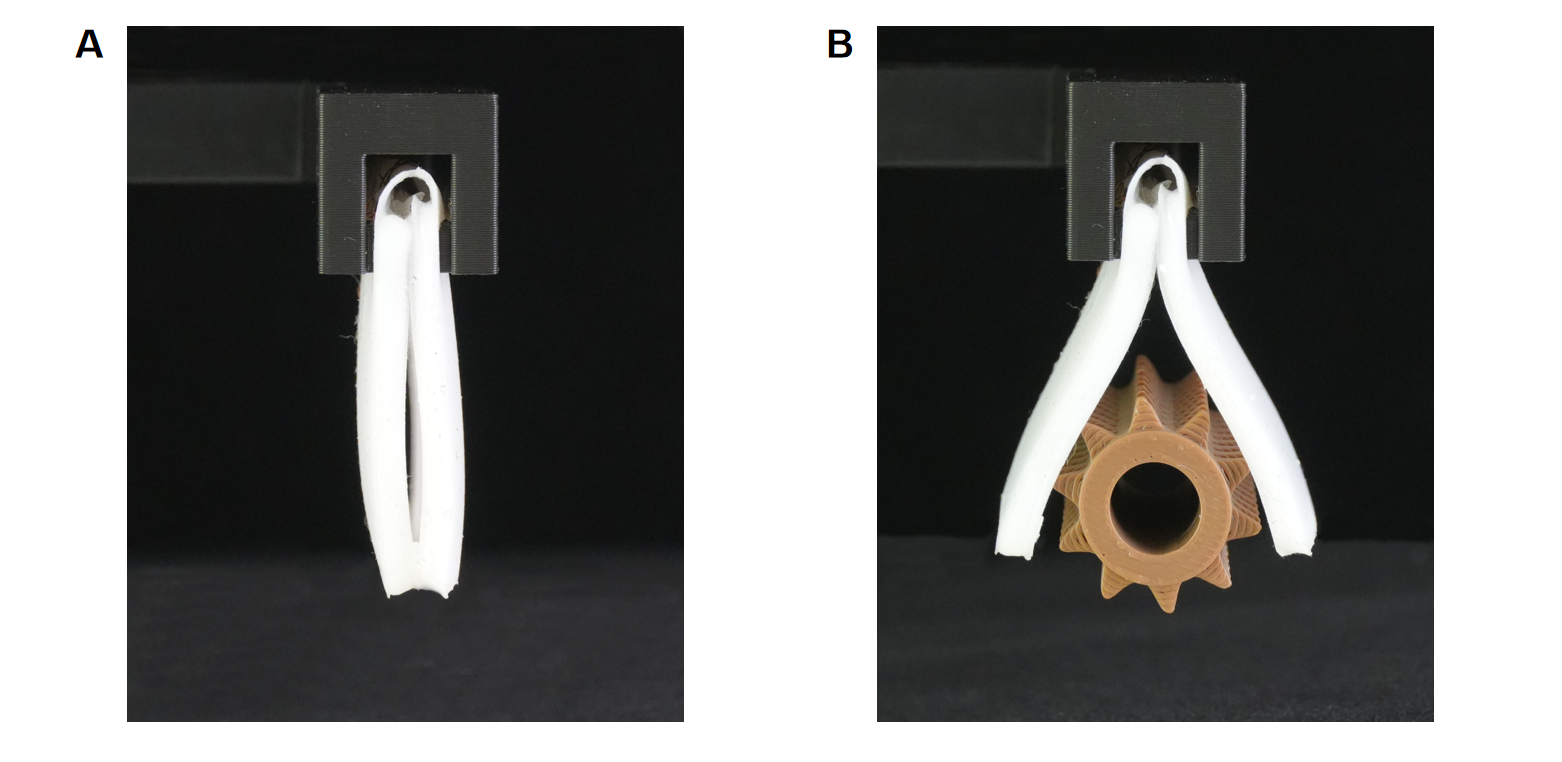


**Fig. S8.** A normally closed gripper. (A) The natural state. (B) The object-grasping state (no current applied).


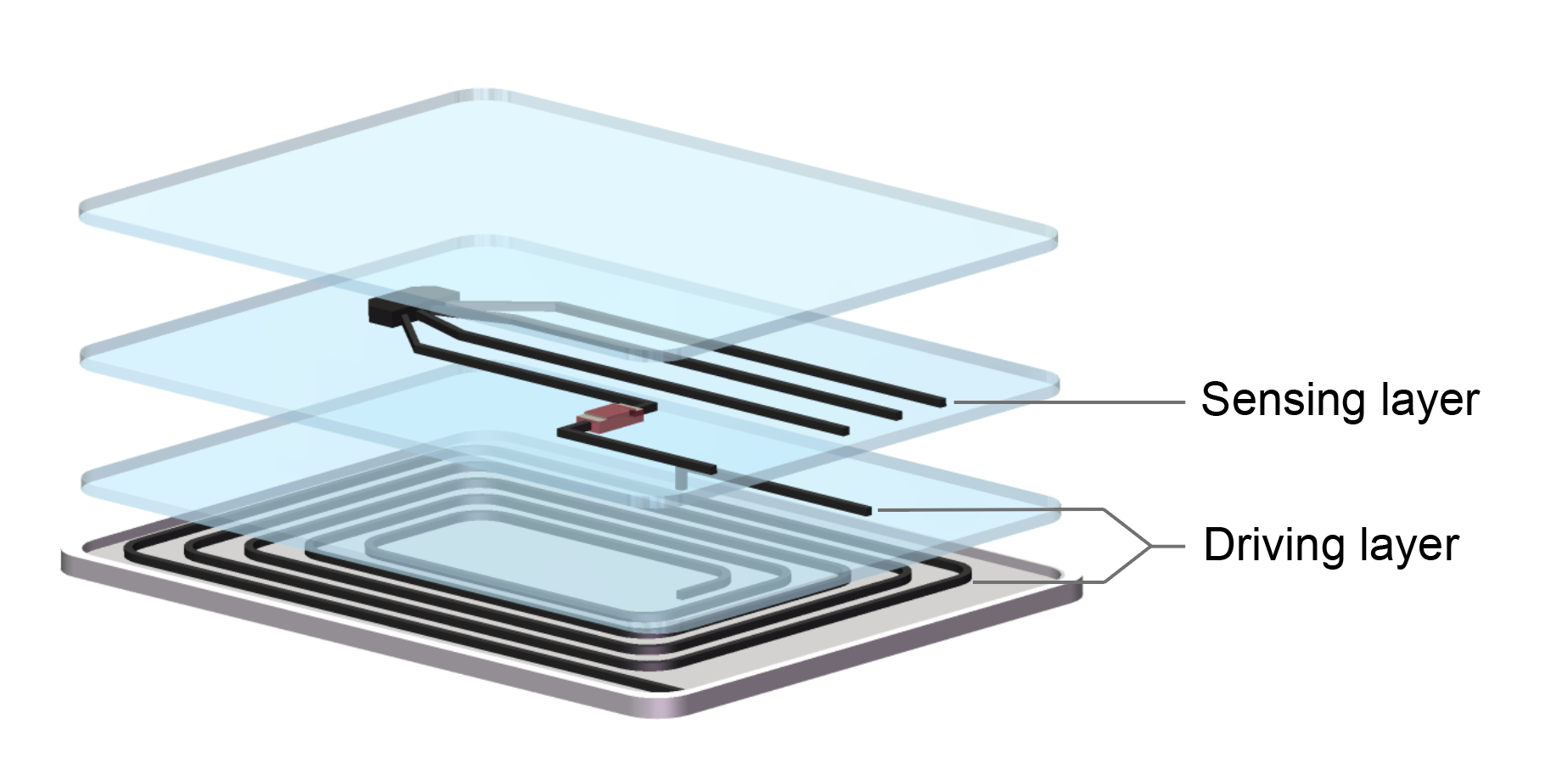


**Fig. S9.** Exploded view of the 3D-printed SEMA with two functional electronic components in the sensing layer.


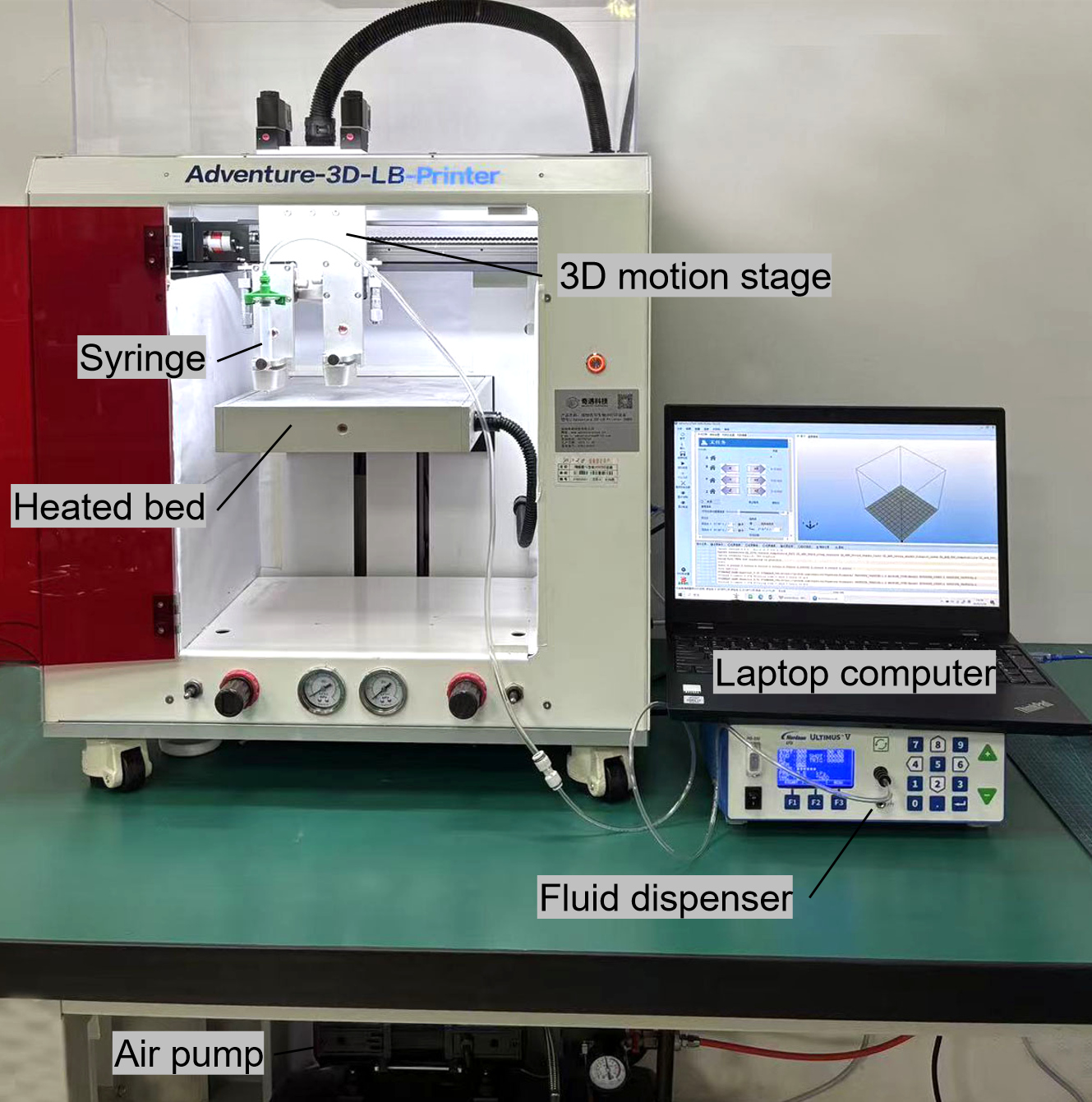


Fig. S10. 3D printing system.


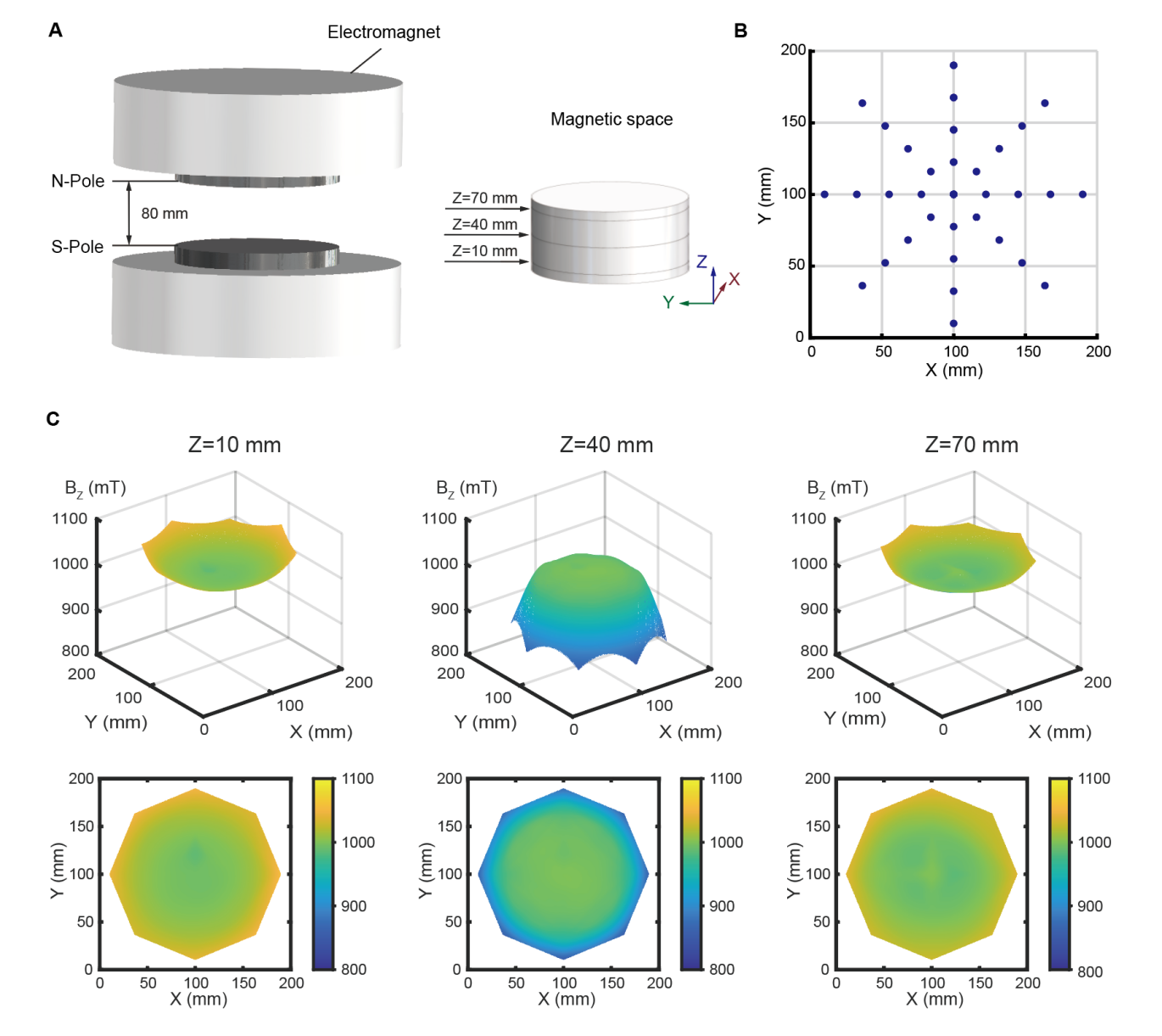


**Fig. S11.** Magnetic field characterization of the vertical electromagnet. (A) Schematic of the vertical electromagnet with a pole face diameter of 200 mm and an operating air gap of 80 mm. The measurement planes are defined at Z = 10, 40, and 70 mm within the magnetic space. (B) Sampling grid showing 33 measurement points on each plane. (C) Magnetic field distributions at Z = 10, 40, and 70 mm, reconstructed from cubic interpolation of the measured data and shown as 3D surface plots and 2D contour maps.


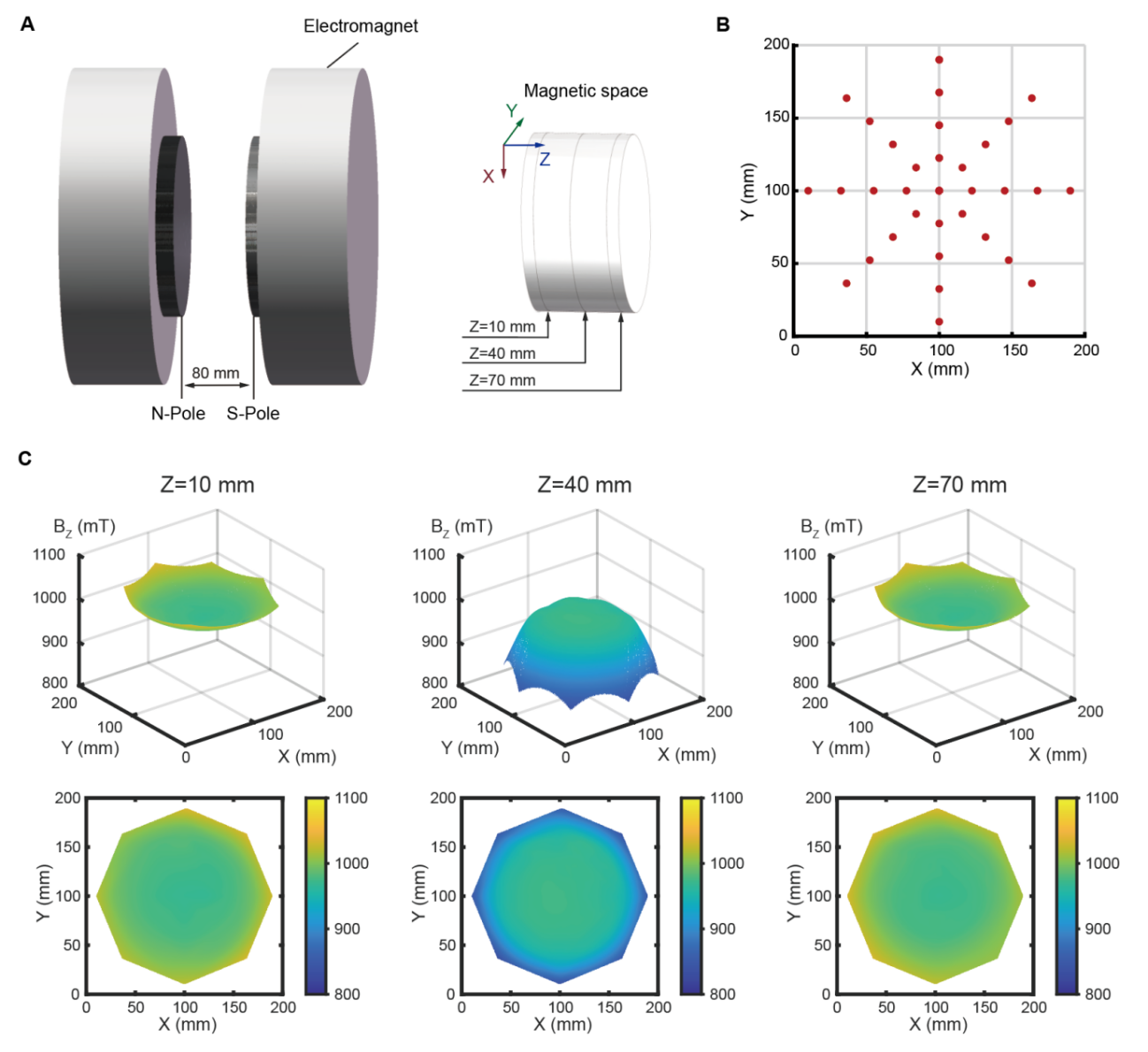


Fig. S12. Magnetic field characterization of the horizontal electromagnet. (A) Schematic of the horizontal electromagnet, oriented perpendicular to the configuration in Fig. S3, with the same pole face diameter and air gap. (B) Sampling grid showing 33 measurement points on each plane. (C) Magnetic field distributions at Z = 10, 40, and 70 mm, obtained using the same measurement and interpolation procedure as in Fig. S3.


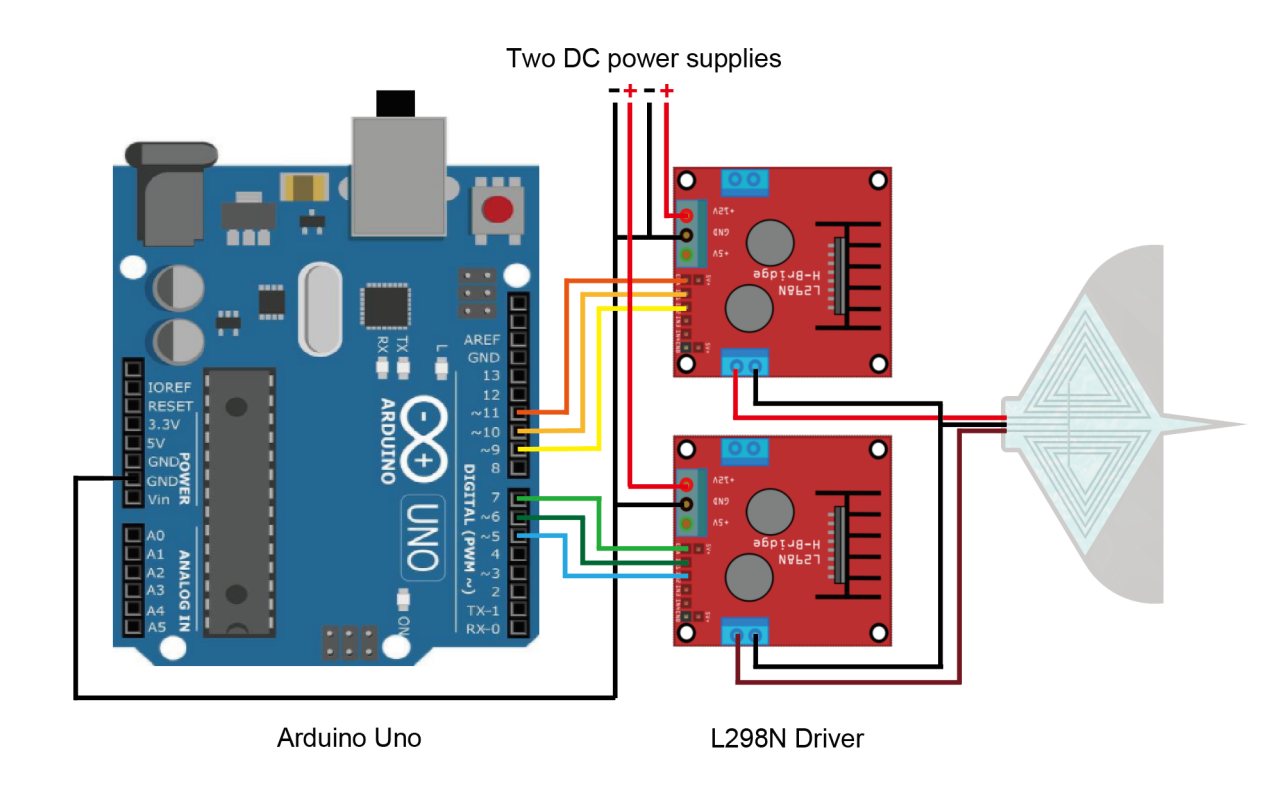


Fig. S13. Control and power system of the manta-like SEMR. Details about this setup are in the section Materials and Methods. The individual components depicted in the image are distributed as part of the Fritzing parts library (https://github.com/fritzing/fritzing-parts), which is licensed under the "Creative Commons Attribution-ShareAlike 3.0 Unported (CC BY-SA 3.0)" license.

Table S1. Comparison of different manufacturing methods of LM-elastomer based soft robots and stretchable electronics.


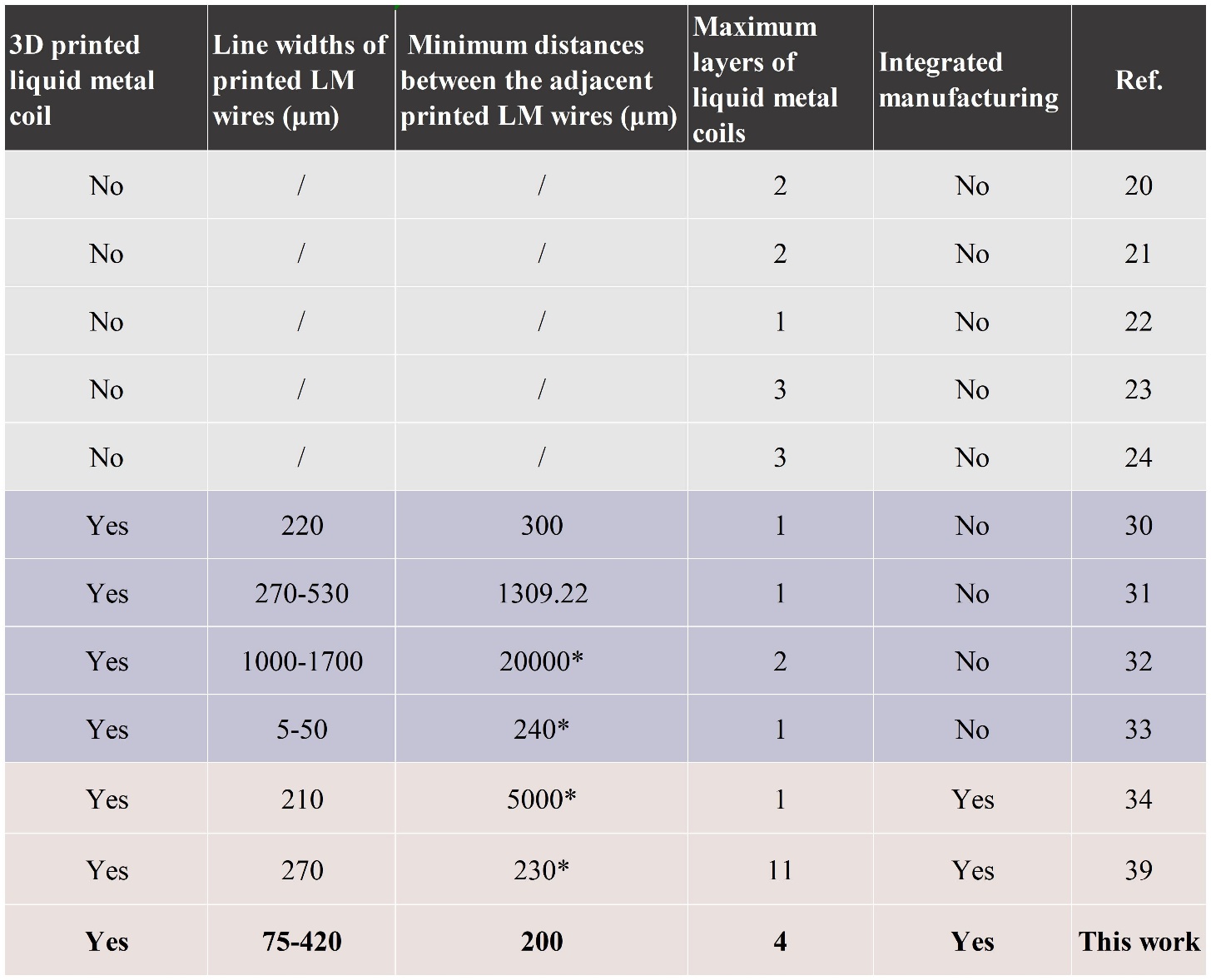


* means that the data is not directly stated in the article and is indirectly obtained from the cases given in the article, and it may not be the technical limit.

Table S2. Printing parameters used for fabricating demos.


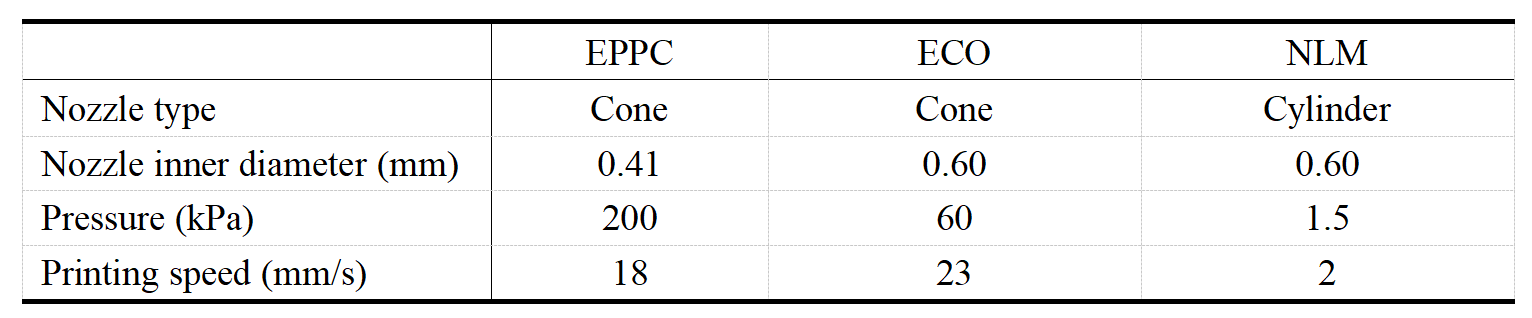


It should be noted that the air pressures mentioned in the article are control values at the ink surface. During printing, the gradual reduction in syringe volumes results in decreases in pressure at the nozzles.

**Table S3.** Printing and curing times of the square-shaped SEMA with a double-layer coil.
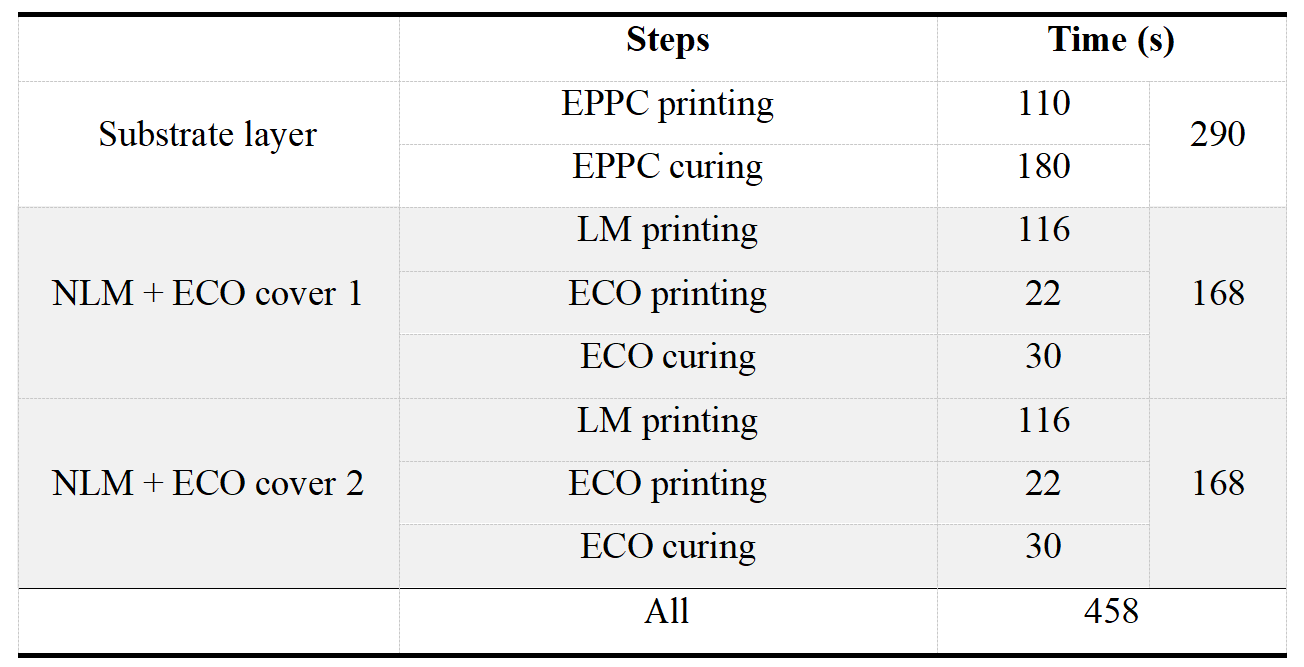


**Table S4.** Comparative table of swimming speed among soft swimming robots.

| Name | Actuation | Max. Speed  (body length/s) | Ref. |
| --- | --- | --- | --- |
| A bionic piezoelectric jellyfish | Piezoelectric actuation | 0.10 | (*3*) |
| A biomimetic muscle actuated soft fish | Shape memory alloy actuation | 0.11 | (*4*) |
| An eel-Inspired soft robot | Pneumatic actuation | 0.36 | (*5*) |
| A soft robotic fish with hydraulic variable stiffness | Electro-Hydraulic actuation | 0.63 | (*6*) |
| A manta-like soft robot | Electromagnetic actuation | 0.66 | This work |
| A soft robotic fish actuated by bionic muscle | Dielectric elastomer actuation | 0.76 | (*7*) |
| An ocyropsis-inspired soft robot | Electro-hydraulic actuation | 1.10 | (*8*) |

**Movie S1. Multi-material printing process**

Multi-material 3D printing of a square-shaped SEMA with a double-layer coil.

**Movie S2. Stretchability of NLM wire embedded in elastomer**

An LED is connected in series with the printed NLM wire. As the wire is stretched from its original length to 200% strain at 2 mm/s, the LED remains lit, demonstrating the high stretchability of the NLM-elastomer composite.

**Movie S3. Cyclic driving test of 3D-printed two-layer SEMAs**The SEMA maintains normal operation after 200,000 cycles.

**Movie S4. Force output comparison of four-layer vs. single-layer NLM-coil SEMAs**

The top sides of the SEMAs are connected to weights via fixed pulleys. Force output comparison of four-layer and single-layer NLM-coil SEMAs under a 1 T magnetic field with a 1 A current.

**Movie S5. Responsiveness of self-sensing SEMAs**In a 1 T magnetic field, the resistance of the sensing layer responds dynamically to the driving stimuli and stabilizes within 1.5 s.

**Movie S6. Sensing capability test of a sensor-integrated soft gripper**

We performed grasping experiments with and without a load. In a 1 T vertical magnetic field, the soft gripper closes under the action of a 1 A driving current and subsequently moves along a prescribed path, with the sensing signal reflecting its operating state.

**Movie S7. A swimming manta-like SEMR**

The 3D-printed manta-like SEMR achieves an average swimming speed of 29 mm/s in water under a 1 T magnetic field driven by a ±0.6 A current at a frequency of 3.3 Hz.

**Movie S8. Mechanical robustness test of SEMA integrated with electronics**The 3D-printed SEMA integrated with electronics remains fully functional after undergoing stretching, repeated bending, and cyclic twisting, verifying its outstanding mechanical robustness.

**REFERENCES**

1. M. D. Bartlett, S. W. Case, A. J. Kinloch, D. A. Dillard, Peel tests for quantifying adhesion and toughness: A review. *Progress in Materials Science* **137**, 101086 (2023).

2. T. Yin, G. Zhang, S. Qu, Z. Suo, Peel of elastomers of various thicknesses and widths. *Extreme Mechanics Letters* **46**, 101325 (2021).

3. J. Xing, W. Jin, K. Yang, I. Howard, A bionic piezoelectric robotic jellyfish with a large deformation flexure hinge. *IEEE Transactions on Industrial Electronics* **70**, 12596–12605 (2023).

4. D. Aragaki, T. Nishimura, R. Sato, A. Ming, Biomimetic soft underwater robot inspired by the red muscle and tendon structure of fish. *Biomimetics* **8**, 133 (2023).

5. D. Q. Nguyen, V. A. Ho, Anguilliform swimming performance of an eel-inspired soft robot. *Soft Robotics* **9**, 425–439 (2022).

6. I. Ju, D. Yun, Hydraulic variable stiffness mechanism for swimming locomotion optimization of soft robotic fish. *Ocean Engineering* **286**, 115551 (2023).

7. R. Wang, C. Zhang, Y. Zhang, L. Yang, W. Tan, H. Qin, W. Feifei, L. Liu, Fast-swimming soft robotic fish actuated by bionic muscle. *Soft robotics* **11** (2024).

8. Z. Ye, G. Yang, H. Dai, Y. Gan, Y. Jian, K. Xu, M. J. Deen, J. Xia, N. Tian, Y. Yang, H. Yang, C. Zhang, Ocyropsis-inspired fast-swimming transparent soft robots. *Advanced Functional Materials* **35**, 2421522 (2025).
